# Vaccines based on the fusion protein consensus sequence protect Syrian hamsters from Nipah virus infection

**DOI:** 10.1172/jci.insight.175461

**Published:** 2023-12-08

**Authors:** Mingqing Lu, Yanfeng Yao, Hang Liu, Xuekai Zhang, Xuejie Li, Yuanhua Liu, Yun Peng, Tong Chen, Yun Sun, Ge Gao, Miaoyu Chen, Jiaxuan Zhao, XiaoYu Zhang, Chunhong Yin, Weiwei Guo, Peipei Yang, Xue Hu, Juhong Rao, Entao Li, Gary Wong, Zhiming Yuan, Sandra Chiu, Chao Shan, Jiaming Lan

**Affiliations:** 1 State Key Laboratory of Virology, Wuhan Institute of Virology, Chinese Academy of Sciences, Wuhan, China.; 2 University of the Chinese Academy of Sciences, Beijing, China.; 3 Center for Biosafety Mega-Science, Wuhan Institute of Virology, Chinese Academy of Sciences, Wuhan, China.; 4 CAS Key Laboratory of Molecular Virology & Immunology, Shanghai Institute of Immunity and Infection Chinese Academy of Sciences, Shanghai, China.; 5 Institute of Neuroscience, State Key Laboratory of Neuroscience, Key Laboratory of Primate Neurobiology, Center for Excellence in Brain Science and Intelligence Technology, Chinese Academy of Sciences, Shanghai, China.; 6 College of Life Sciences, Nanjing Normal University, Nanjing, China.; 7 Division of Life Sciences and Medicine, University of Science and Technology of China, Hefei, China.; 8 Hubei Jiangxia Laboratory, Wuhan, China.

**Keywords:** Vaccines, Virology, Adaptive immunity

## Abstract

Nipah virus (NiV), a bat-borne paramyxovirus, results in neurological and respiratory diseases with high mortality in humans and animals. Developing vaccines is crucial for fighting these diseases. Previously, only a few studies focused on the fusion (F) protein alone as the immunogen. Numerous NiV strains have been identified, including 2 representative strains from Malaysia (NiV-M) and Bangladesh (NiV-B), which differ significantly from each other. In this study, an F protein sequence with the potential to prevent different NiV strain infections was designed by bioinformatics analysis after an in-depth study of NiV sequences in GenBank. Then, a chimpanzee adenoviral vector vaccine and a DNA vaccine were developed. High levels of immune responses were detected after AdC68-F, pVAX1-F, and a prime-boost strategy (pVAX1-F/AdC68-F) in mice. After high titers of humoral responses were induced, the hamsters were challenged by the lethal NiV-M and NiV-B strains separately. The vaccinated hamsters did not show any clinical signs and survived 21 days after infection with either strain of NiV, and no virus was detected in different tissues. These results indicate that the vaccines provided complete protection against representative strains of NiV infection and have the potential to be developed as a broad-spectrum vaccine for human use.

## Graphical abstract

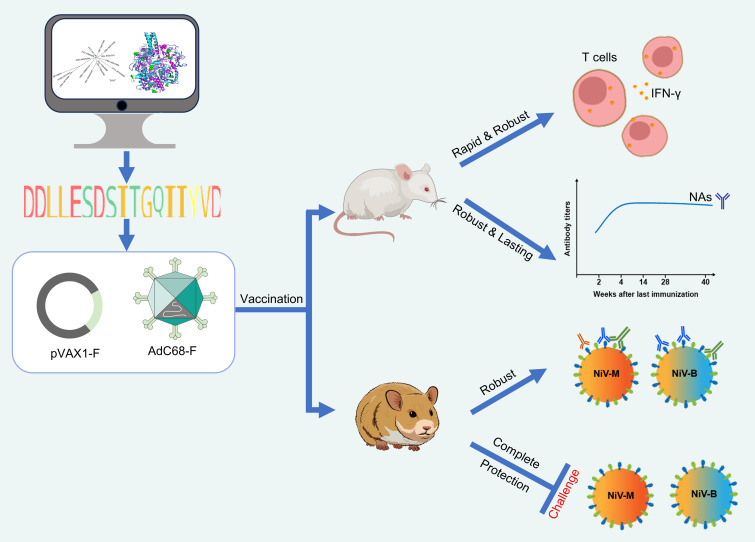

## Introduction

Nipah virus (NiV) is a single-stranded negative-sense RNA virus belonging to the *Henipavirus* genus of the family *Paramyxoviridae*. It is closely related to Hendra viruses of other *Henipavirus* species. It was first discovered in Kampung Sungai Nipah in Malaysia in 1998–1999 and named accordingly (1). The main reservoir of NiV is fruit bats in the genus *Pteropus*, which can transmit the virus to humans directly or through intermediate hosts such as pigs or horses (2–4). According to the World Health Organization (WHO), as of now, there have been 25 reported outbreaks of NiV in South Asia, with 429 cases and approximately 307 deaths (5–7). Malaysia, Bangladesh, India, the Philippines, and Singapore are among the common outbreak areas of NiV (1, 8–11). Severe cases of NiV infection in humans present as a respiratory disease, encephalitis, or a combination of both (12). Due to its easy transmission, rapid onset, severe symptoms, high mortality rate, and lack of effective countermeasures, NiV is classified as a category C priority pathogen and a biosafety level 4 (BSL-4) agent by the Centers for Disease Control and Prevention and the National Institute of Allergy and Infectious Diseases (13). It is also considered to be a bioterrorism and agroterrorism threat (14, 15). In addition, it has been listed as one of the top 10 priority diseases by the WHO (16). To protect 2 billion people currently living in regions in which NiV is endemic or in which the *Pteropus* fruit bat reservoir is commonly found (17), there is an urgent need for the development of NiV vaccines for human and animal use.

It was found that in Malaysia, NiV primarily spreads to humans through pigs as intermediate hosts, resulting in human-to-human transmission rarely, and the mortality rate of NiV disease is relatively low, approximately 40% (1, 18). However, in Bangladesh, NiV mainly spreads to humans directly, causing extensive human-to-human transmission and a higher mortality rate of approximately 70% to 90% (19). Therefore, there are 2 divergent NiV strains, named Malaysian (NiV-M) and Bangladeshi (NiV-B). Their genomes share 91.8% sequence identity (20). In addition, multiple strains of NiV have been isolated in India, Thailand, the Philippines, Singapore, and other countries (21). As of now, approximately 330 NiV sequences have been publicly available on the GenBank website. Based on the experience of developing vaccines for RNA viruses that are prone to mutation, such as SARS-CoV-2, influenza virus (22, 23), and partial heterologous protection of NiV vaccines (24), it is of great scientific and strategic significance to develop vaccines that have the potential to protect against existing and potential new strains of NiV. Currently, consensus sequence–based vaccines are being developed to meet these demands (25–27).

NiV is enveloped with 2 important glycoproteins, attachment (G) protein and fusion (F) protein. The former mediates the binding of the virus to receptors on susceptible cells, while the latter mediates the fusion of the virus envelope with the host cell membrane (28). Studies have found that the G protein contains the main neutralizing antigenic epitopes (29, 30), making it the most commonly targeted antigen for vaccine development. Currently, various NiV vaccines targeting the G protein, such as recombinant protein vaccines (31), viral vector vaccines (32–34), and mRNA vaccines (35, 36), are under development. The only human monoclonal antibody that has been evaluated for NiV protection studies in the African green monkey model, as well as having undergone a phase I clinical study, m102.4 (37), also targets the G protein of *Henipavirus*. The F protein has also been investigated for NiV vaccine development (24, 35, 38), but research on it is relatively scarce compared with that on the G protein.

The F protein is synthesized as a precursor F0 that undergoes posttranslational cleavage by host cell cathepsin L within the endosomal compartment during endocytosis and recycling, yielding the fusogenic F1 and F2 subunits that are held together by a disulfide bond (39). As with other paramyxoviruses, the F protein of NiV undergoes a significant refolding transition during the viral entry process, which results in the 2 different conformations, pre-F and post-F. Furthermore, previous research has shown that a pre-F–stabilized subunit vaccine of respiratory syncytial virus, another member of *Paramyxoviridae*, elicits a broader and more potent antibody response than a post-F vaccine (40).

Based on the publicly available NiV sequences on the GenBank website, this study designed a consensus sequence vaccine targeting the NiV F protein. Chimpanzee adenoviral vectors and plasmids were used as carriers, and the efficiency of the vaccines was investigated individually and in combination as a NiV vaccine, with the aim of inducing immune responses and achieving immune protection in proper animal models of NiV.

## Results

### The consensus sequence of the F protein was generated and analyzed by the bioinformatics method.

**Figure 1:**
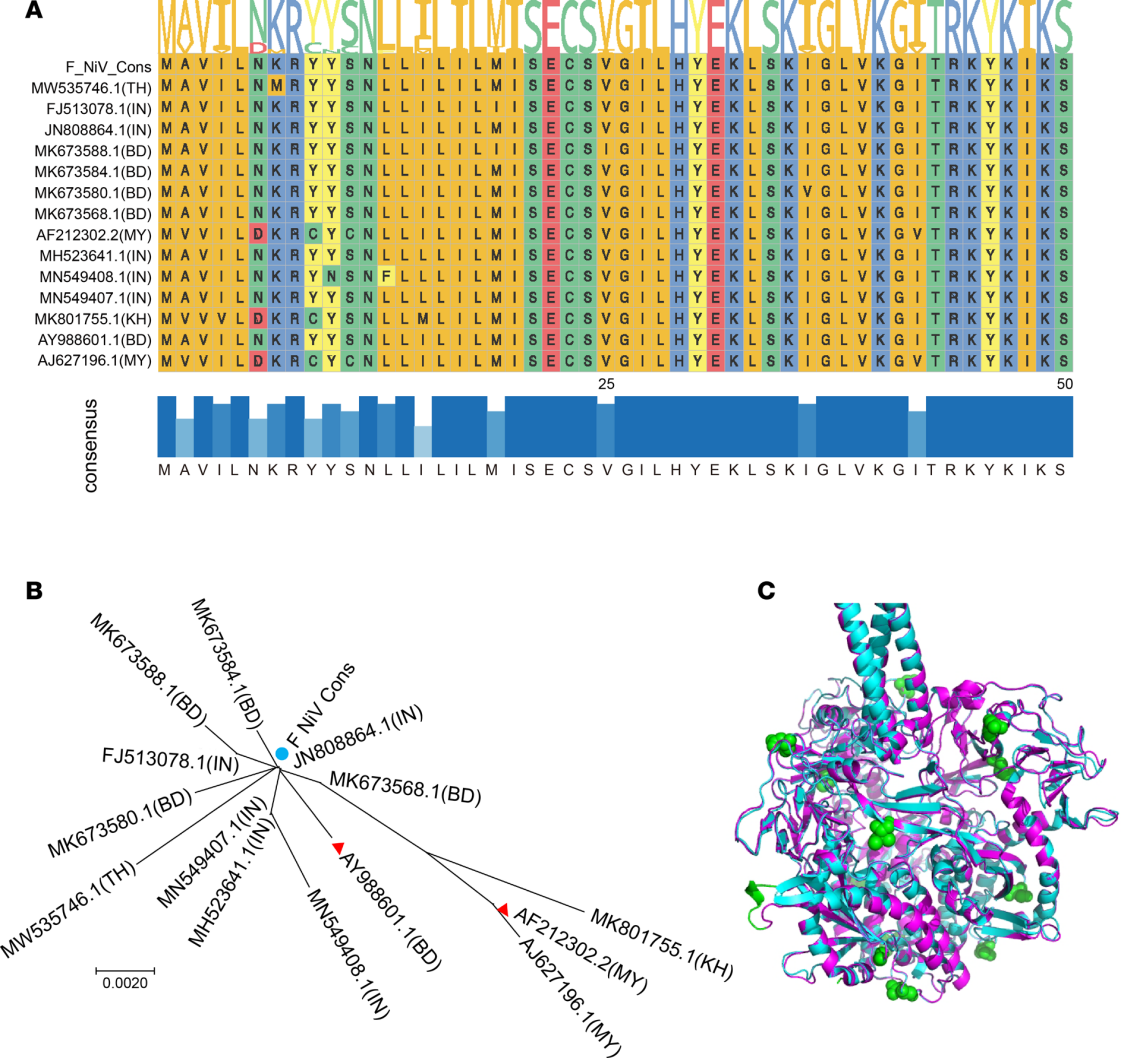
The bioinformatic characteristics of the consensus sequence of the F protein. (**A**) Partial consensus sequences (1–50 aa) are shown in the vaccine study by ggmsa function analysis of the amino acid consistency results obtained in the R software package. (**B**) The phylogenetic tree results of the consensus sequence of F with other representative sequences of GenBank. The consensus sequences in the study are shown as blue circles, and the challenge virus strains are shown as triangles. The strain label is derived from GenBank. The letters in the brackets are the abbreviations of the countries where the viruses were isolated. MY = Malaysia, BD = Bangladesh, IN = India, and KH = Cambodia. (**C**) The 3D structure of the consensus F protein (magenta) was predicted with SWISS-MODEL software. The results are shown superimposed on the consensus sequence with the resolved prefusion conformation F protein (cyan) (PDB: 5EVM).

According to the principle of sequence consensus, we obtained a conserved antigen of the F protein for vaccine development from the NiV sequence in GenBank based on bioinformatics analysis. For clear presentation, conserved sequences and a partial consensus sequence (1–50 aa) are shown in Figure 1A; the remaining sequences are shown in Supplemental Figure 1 (supplemental material available online with this article; https://doi.org/10.1172/jci.insight.175461DS1). The amino acid sequences of the F protein designed in this study covered a frequency range of 76.62%–100% in GenBank, and the coverage frequency of most amino acid sequences was above 95%. To ensure the conservation of the designed F protein sequence in this study, we also conducted an unrooted phylogenetic tree analysis, as shown in Figure 1B. It can be seen that the F protein sequence (shown as a blue circle) is closely related to most (11 of 14) of the representative strains of F. Apart from them, only 3 strains of F were far away from the consensus sequences, one of which with a red asterisk (AF212302.2) was used for the virus challenge experiment. Similarly, the other strain marked with a red asterisk (AY988601.1) of the 11 closely related viruses was also selected for the virus challenge assay. By using the SWISS-Model modeling method, we obtained a 3D structure schematic diagram of the consensus F protein in Figure 1C (marked with magenta), and the structure of the pre-F protein (PDB: 5VEM) was automatically selected as the template by the software. The consistency of the modeled structure between them is approximately 0.60, and the conformational matching degree is 0.82 ± 0.05. The reliability of the modeling is indicated by a global model quality estimate (GMEM) value of approximately 0.76. It was speculated that there is a high probability that the F protein designed in this study also exists in a pre-F homotrimeric conformation.

### Recombinant chimpanzee adenoviral vector vaccine and DNA vaccine based on the consensus sequences of the F protein were successfully developed.

**Figure 2:**
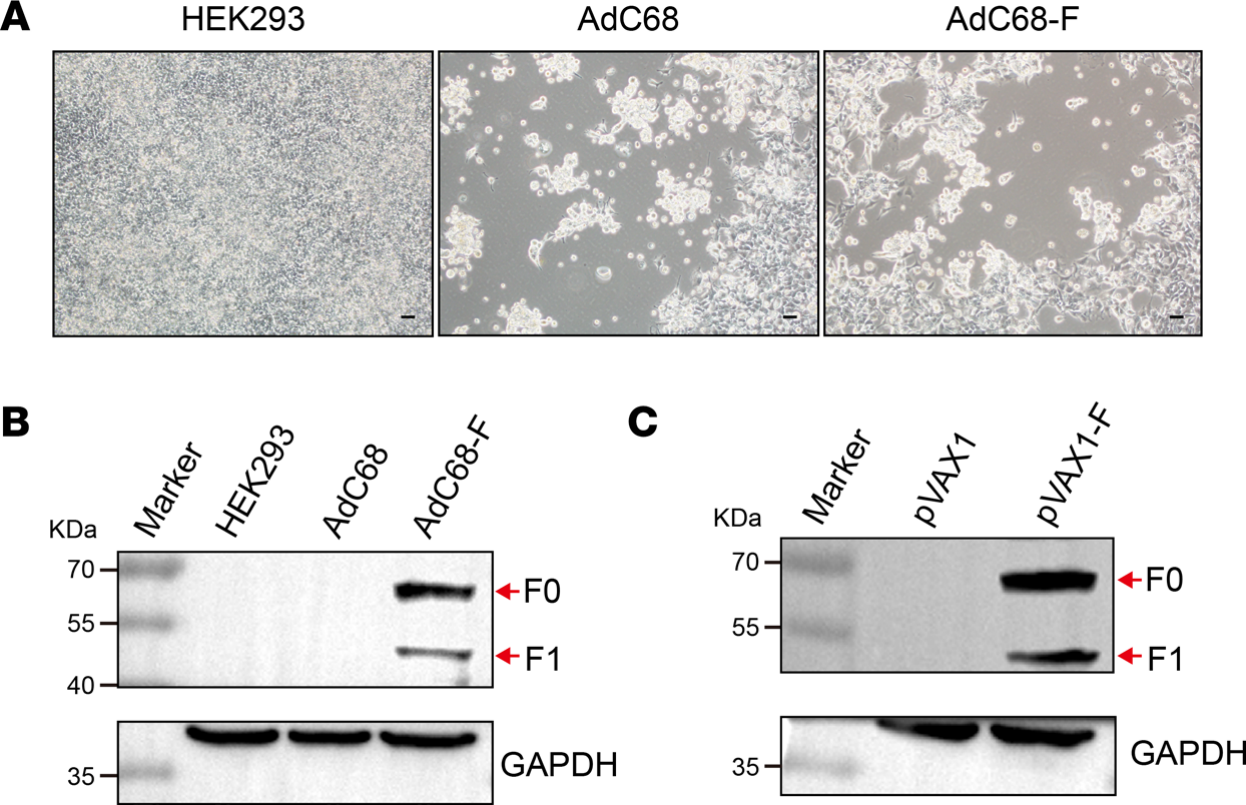
The verification of acquired recombinant AdC68-F and pVAX1-F. (**A**) The recombinant AdC68 and AdC68-F viruses resulted in CPEs in HEK293 cells. HEK293 cells exhibiting CPEs changed from a fibrous to a rounded shape and detached from the plates. Scale bars: 100 μm. (**B** and **C**) The specific bands of F protein were detected by Western blotting of AdC68-F–infected HEK293 cells (**B**) and pVAX1-F–transfected HEK293T cells (**C**). Both F0 (61 kDa) and F1 (49 kDa) could be detected in AdC68-F–infected and pVAX1-F–transfected cells.

Linearized pAdC68 or pAdC68-F plasmids were transfected into HEK293 cells, which resulted in cytopathic effects (CPEs) such as rounded cells and detachment from the plates, 8 to 10 days after transfection (Figure 2A). Equivalent CPEs were seen for both the backbone AdC68 plasmid and AdC68-F, representing the effects of rescued recombinant virus. The suspected rescued recombinant virus was collected and used to infect HEK293 cells. After CPEs were evident in 80% of the plates, cells were harvested and analyzed by Western blotting with F-specific antibodies. The recombinant AdC68-F–infected cells showed F-specific bands at approximately 61 kDa (F0) and 49 kDa (F1) (Figure 2B). Similarly, the expression of F protein was confirmed by Western blotting 48 hours after transfection of plasmid pVAX1 or pVAX1-F into HEK293T cells (Figure 2C). These results demonstrated that the recombinant chimpanzee adenoviral vector vaccine AdC68-F and DNA vaccine pVAX1-F based on the F consensus sequence were successfully developed.

### The vaccine regimens of AdC68-F and pVAX1-F induced high levels of humoral and cellular responses in mice.

**Figure 3:**
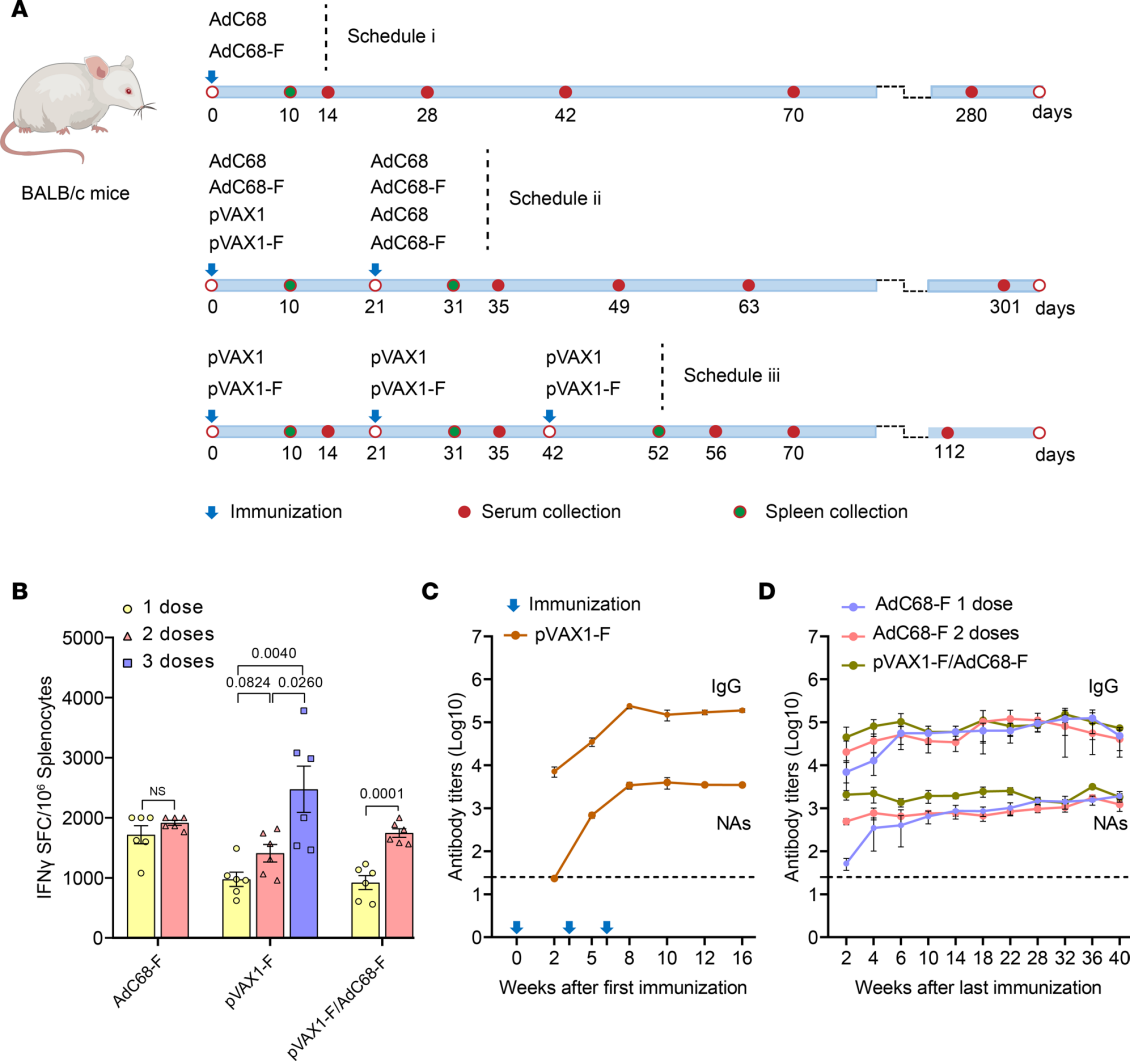
The immune schedules and immune responses to the vaccine regimens induced in mice. (**A**) The immune schedules of the mice. The mice were immunized once (i), twice (ii), or 3 times (iii). The sera and spleen tissues were harvested at different times for humoral and cellular immune response detection. (**B**) F-specific cellular immune responses were detected by ELISpot. Ten days after each immunization, the splenocytes of 6 mice in each vaccine group were collected (control group *n* = 3), and the number of T lymphocytes that could secrete IFN-γ is shown on the *y* axis under the stimulation with F peptides. SFC, spot-forming cells. *P* values are indicated in the graph. (**C**) The F-specific IgG antibodies and NAs induced by the pVAX1-F vaccine regimens were detected by ELISA and pseudovirus neutralization assay. Sera were collected at different times (2, 5, 8, 10, and 16 weeks) after the first immunization from each group. The dashed line represents the lower limit of detection (20 × dilution) (control group *n* = 3, vaccination group *n* = 6). (**D**) The F-specific IgG antibodies and NAs detected by ELISA and the pseudovirus neutralization assay in AdC68-F and pVAX1-F/AdC68-F vaccine regimens. The sera were collected at different times (2, 4, 6, 10, 14, 18, 22, 28, 32, 36, and 40 weeks) after the last immunization (control group *n* = 3, vaccination group *n* = 6). The F-specific IgG antibodies (IgG, upper) and neutralization antibodies (NAs, lower) are shown. The dashed line represents the lower limit of detection (25 × dilution). Data are shown as the mean ± SEM. Statistical significance was determined by 1-way ANOVA with Tukey’s multiple-comparison test. NS, not significant.

The mice were immunized and grouped as shown in Figure 3A and Table 1. Ten days after each vaccination, 6 mice were randomly euthanized in each group. Splenocytes were separated aseptically, and T lymphocytes secreting IFN-γ were detected after stimulation with F-protein–specific antigenic peptides. As shown in Figure 3B, the AdC68-F vaccine induced a high level of F protein–specific cellular immune response in mice after a single injection, with approximately 1716 spot-forming cells (SFCs), which increased to 1916 SFCs when the mice received a boost vaccination. However, there was no significant difference between the 2 groups (*P* > 0.05). After a single injection of the pVAX1-F vaccine, a certain level of F-protein–specific cellular immune response was induced, with approximately 976 SFCs, and the level of cellular immune response increased with increasing immunization times. SFCs were increased to 1410 and 2474, respectively, after the mice received boost immunization once or twice. However, there was no significant difference between the first boost immunization and the initial immunization (*P* = 0.0824). After the second boost immunization, the level of the cellular immune response was significantly increased, and the *P* values between the first immunization and the first boost immunization were 0.004 and 0.026, respectively. Similarly, the level of T cell immune response was also significantly improved after the first immunization with pVAX1-F and 1 dose of AdC68-F boost (*P* = 0.0001). The F-specific T cell immune response was not detected in mice immunized with empty vectors.

To understand the humoral immune responses induced by the pVAX1-F vaccine, sera were collected at different times (2, 5, 8, 10, 12, and 16 weeks) after the first immunization, and the F-specific IgG antibodies and neutralization antibodies (NAs) induced by the vaccine regimens were detected by ELISA and pseudovirus neutralization assay. As shown in Figure 3C, a single injection of pVAX1-F induced a high level of IgG antibodies, although most of them had no neutralization activities. The titers of IgG antibodies increased gradually with the increase in immunization times, reaching 238,933 at 2 weeks after the third immunization and remaining at a high titer level until 16 weeks (without monitoring for longer times). The NA titers started to rise 2 weeks after the first vaccination and significantly increased to a titer of 3411 at 2 weeks after the second vaccination. The NA titer reached a peak of 3966 at 4 weeks after the third vaccination and continued until 10 weeks after the last vaccination. The NA titers were not monitored beyond that time point.

The sera of 6 mice in each group were collected at different times (2, 4, 6, 10, 14, 18, 22, 28, 32, 36, and 40 weeks) after the last immunization in the AdC68-F and pVAX1-F/AdC68-F vaccine regimen groups. The F-specific IgG antibodies and NAs were detected by ELISA and the pseudovirus neutralization assay. As shown in Figure 3D, 2 weeks after AdC68-F vaccination, the F-specific IgG antibody titers were as high as 6933, and the antibody titers gradually increased with time. Six weeks after AdC68-F immunization, the IgG antibody titers reached 51,200 and remained at a high titer level until 40 weeks (without monitoring for longer times) after immunization, with no sign of decrease. After a boost, the AdC68-F vaccination induced an F-specific IgG antibody titer of 20,267 (2 weeks after the boost immunization), which is higher than that induced by a single immunization. However, this F-specific IgG preponderance no longer existed 6 weeks after the boost immunization, when the titers reached 51,200. Furthermore, the high level of IgG showed no sign of decreasing at the end of the detection period. The prime-boost immunization of pVAX1-F/AdC68-F regimens induced the highest level of IgG when it was detected 2 weeks after the immunization and reached as high as 44,800. As before, this preponderance did not remain as time passed. In summary, all 4 vaccines induced high-level and lasting F-specific IgG antibodies. However, the difference among them was not significant over time. NAs were consistent with the IgG antibodies described above. All 4 vaccines induced high-level and lasting NAs. Among them, the pVAX1-F/AdC68-F regimens induced the highest level of NAs. It is worth mentioning that the titer of NAs reached 2057 with the pVAX1-F/AdC68-F regimen after 2 weeks of immunization, while the other 2 groups achieved 493 (2 doses) and 52 (1 dose). Although both of the other immunizations induced higher levels of NAs as time passed, the pVAX1-F/AdC68-F regimen almost completely maintained their preponderance. The F-specific IgG antibodies and NAs were not detected in mice immunized with empty vectors.

### The vaccine regimens of AdC68-F and pVAX1-F induced high levels of F-specific IgG and neutralization antibodies in Syrian hamsters.

**Figure 4:**
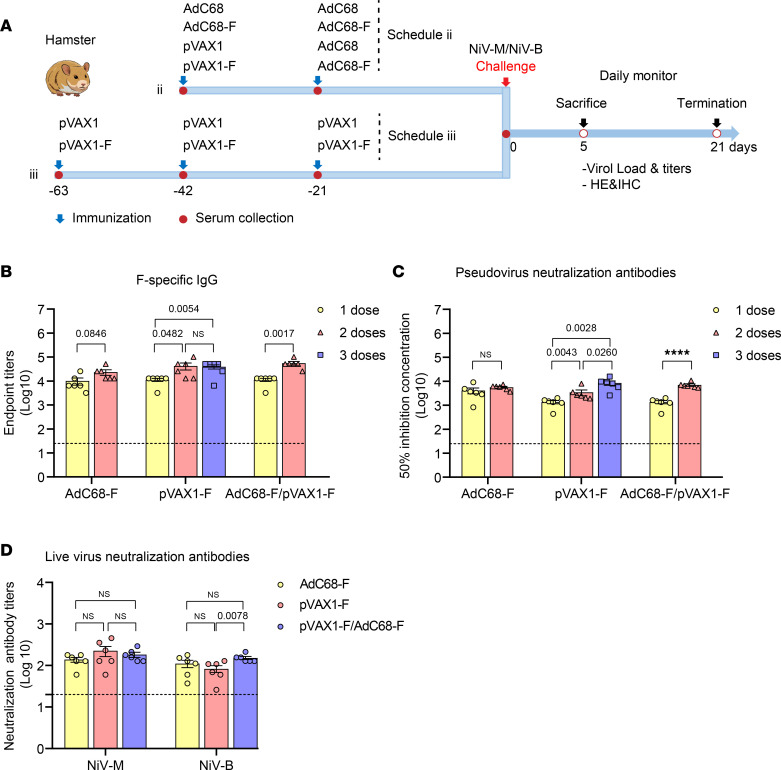
The schedule of the vaccine regimens and the humoral immune response induced in hamsters. (**A**) The schedule of the vaccine regimens conducted in hamsters. After the hamsters received 2 or 3 doses of vaccine immunization, either NiV-M or NiV-B was injected into the hamsters. Subsequently, weight and survival were monitored daily until 21 days after the challenge. Five days after the virus challenge, 6 hamsters of each group were scarified for viral and pathological detection. (**B**) F-specific IgG induced by the vaccine regimens. All 3 vaccine regimens induced high levels of IgG antibodies, and the titers of IgG were elevated with increasing immunization times (*n* = 6). (**C**) The NAs in different vaccine regimen–immunized sera of hamsters by pseudovirus neutralization assay. Consistent with the level of IgG, the vaccine regimens induced high titers of NAs, which increased with increasing immunization time (*n* = 6). (**D**) The NAs in different vaccine regimen–immunized sera of hamsters detected by microneutralization assay of NiV-M and NiV-B. Most of the hamsters (except 1) immunized with the vaccine regimens developed high titers of NAs against NiV-M and NiV-B at the same time (*n* = 6). Data are presented as the group mean ± SEM. Statistical significance was determined by 1-way ANOVA with Tukey’s multiple-comparison test (**D**). Two-tailed, unpaired Student’s *t* tests were conducted to compare differences between 2 experimental groups (**B** and **C**). NS, not significant. ****P < 0.0001.

Following the schedule outlined in Figure 4A and Table 2, the AdC68-F vaccine was able to induce high levels of IgG antibodies in Syrian hamsters after a single intramuscular (i.m.) injection, with antibody titers reaching 10,133 in Figure 4B. Upon homologous boosting, IgG antibody levels were slightly increased, but no statistically significant differences were observed (*P* = 0.0846). The IgG antibody level of the pVAX1-F vaccine increased gradually with increasing immunization times. After the third immunization with pVAX1-F, the IgG titers were as high as 39,467, while there was no significant increase (*P* > 0.05) in IgG antibody levels compared to the second immunization. The difference between the first and second immunization was significant (*P* = 0.0482). Similarly, when the initial immunization with pVAX1-F was followed by heterologous boosting with AdC68-F, there was a significant increase (*P* = 0.0017) in IgG antibody levels in hamsters, with titers of 55,467. F-specific IgG antibody was not detected in the sera of any hamsters immunized with empty vectors.

The results of the pseudovirus neutralization assay showed that the neutralizing antibody levels in the sera of immunized hamsters were consistent with the IgG antibody titers described above. After a single immunization with AdC68-F vaccines, titers as high as 4112 were induced in hamsters (Figure 4C), which were slightly increased (as high as 5606) upon homologous boosting, but no statistically significant difference was observed (*P* > 0.05). The level of neutralizing antibody induced by the pVAX1-F vaccine increased gradually from 1350, to 3428, and to 8540 with increasing immunization times. After heterologous boosting with AdC68-F following initial immunization with pVAX1-F, neutralizing antibody levels were significantly increased (*P* < 0.0001) in hamsters from 1350 to 6992. No NAs were detected in the sera of hamsters immunized with empty vectors.

The sera of hamsters obtained 3 weeks after the last immunization with different vaccine regimens were incubated with live NiV-M and NiV-B viruses to assess the neutralizing ability of the vaccine-induced antibodies. Except for 1 hamster in the pVAX1-F vaccine group, which induced NAs near the lower limit for NiV-B, almost all of the vaccine-immunized hamster sera were able to neutralize both strains of NiV, as shown in Figure 4D. Except for the difference between the pVAX1-F and pVAX1-F/AdC68-F groups’ response to NiV-B (*P* = 0.0078), there were no significant differences observed among the different vaccine groups in Figure 4D. In general, the neutralizing capacity of the vaccine-induced antibodies against NiV-M was slightly stronger than that against NiV-B. No neutralizing capacity against NiV-M or NiV-B was detected in the sera of hamsters immunized with empty vectors. Overall, the vaccine regimens induced high levels of IgG and neutralizing antibodies in hamsters against NiV-M and NiV-B at the same time.

### All of the vaccine regimen–immunized hamsters survived lethal NiV-M and NiV-B challenge.

**Figure 5:**
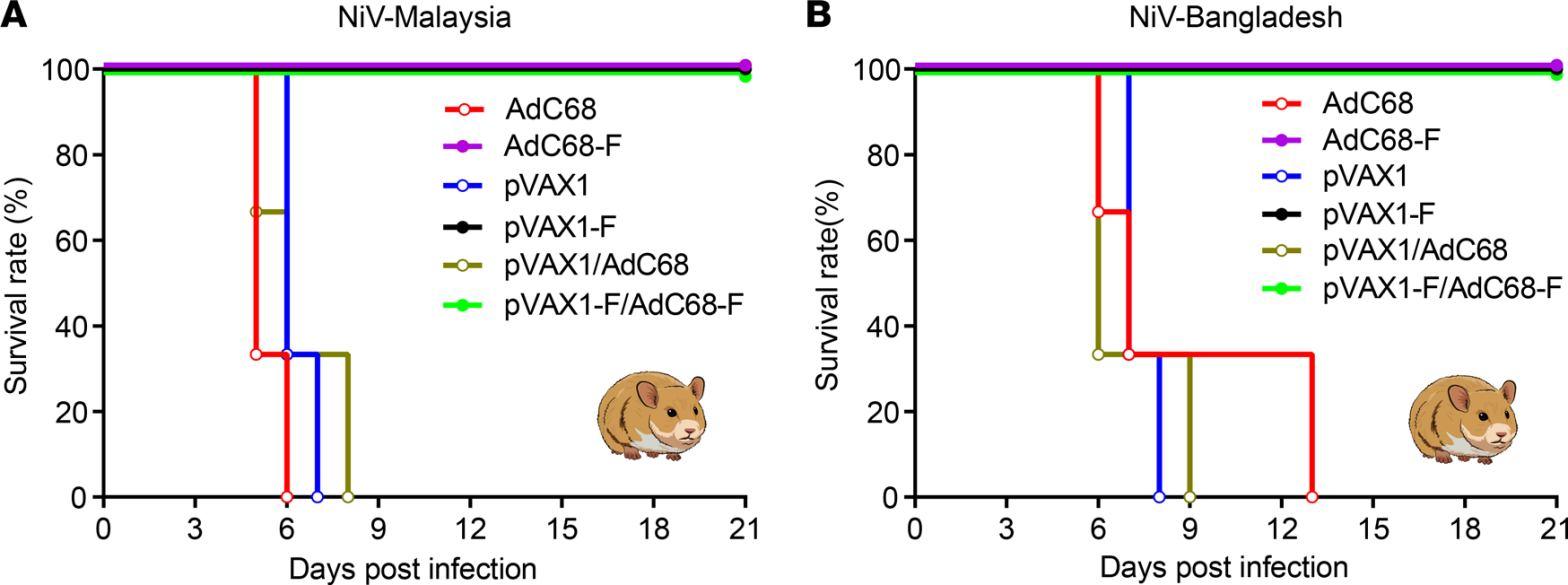
The survival rate of vaccine regimen–immunized hamsters after challenge with NiV-M and NiV-B. (**A**) After challenge with 1000 LD_50_ of NiV-M, all of the vaccine regimen–immunized hamsters (*n* = 6) survived the endpoint of the experiment, while the control animals (*n* = 3) died 5–8 days after the challenge. (**B**) Similarly, 6 to 13 days after the NiV-B challenge, all of the hamsters in the empty vector–immunized groups (*n* = 3) died, while the vaccine regimen–immunized hamsters (*n* = 6) did not show any sign of disease and survived 21 days after the infection.

Two weeks after the last vaccination, hamsters were transported from an animal BSL-2 (ABSL-2) to an ABSL-4 facility. After a 1-week adaptation, 1000 LD_50_ of the NiV-M or NiV-B strain was intraperitoneally (i.p.) injected into Syrian hamsters separately. The animals were observed daily for food and water intake, disease symptoms, changes in body weight, and survival status until 21 days after the virus challenge. Four days after NiV-M infection, AdC68 or pVAX1 vector–immunized hamsters showed decreased appetite, piloerection, arched backs, and reduced activity when they were at rest. Five to 8 days after virus infection, all empty vector–immunized hamsters died from NiV-M infection, while all 18 hamsters in the 3-vaccine regimen–immunized groups did not show any sign of illness and had no significant weight changes (data not shown), and no deaths occurred throughout the experimental period (Figure 5A). Similar clinical symptoms were observed in the empty vector–immunized hamsters infected with NiV-B. Six to 13 days after NiV-B infection, all AdC68 or pVAX1 empty vector–immunized hamsters died from NiV-B infection, whereas no signs of illness or weight loss (data not shown) were observed in the vaccine regimen–immunized hamsters until the endpoint of 21 days after virus infection, and no deaths occurred, as shown in Figure 5B.

### Neither NiV-M nor NiV-B was detected in the tissues of vaccine regimen–immunized hamsters after NiV challenge.

**Figure 6:**
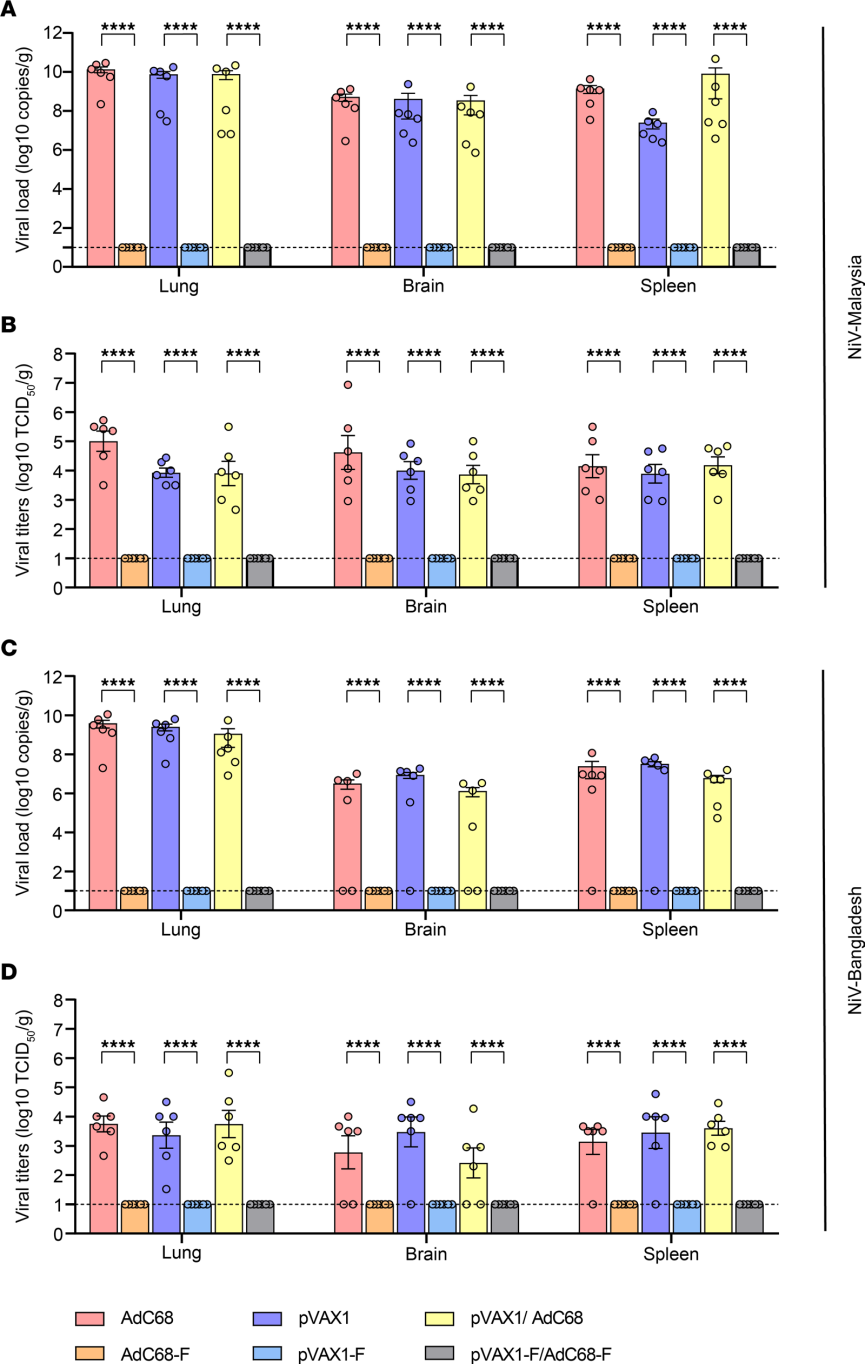
Viral nucleic acid load and viral titers in different tissues of hamsters after challenge with NiV. Five days after NiV-M or NiV-B infection, 6 hamsters were randomly selected and euthanized to collect the lung, brain, and spleen tissues. Real-time PCR and virus titration experiments showed that hamsters of empty vector groups (*n* = 6) infected with NiV-M had a high viral load in the lung, brain, and spleen tissues, while vaccinated hamsters had no viral nucleic acid (**A**) or infectious viral particles (**B**). Similarly, after NiV-B infection, most animals (except for 2 of AdC68 in the brain, 1 of pVAX1 in the brain, 2 of pVAX1/AdC68 in the brain, 1 of AdC68 in the spleen, and 1 of pVAX1 in the spleen) in the empty vector control groups showed high viral load (**C**) and infectious viral particles (**D**) in the lung, brain, and spleen tissues, while the vaccine regimens protected the hamsters against the virus without any viral nucleic acid or infectious viral particles in these tissues. Data are shown as the mean ± SEM. Two-tailed, unpaired Student’s *t* tests were conducted to compare differences between 2 experimental groups. ****P < 0.0001.

Five days after NiV infection, 6 hamsters were randomly selected from each group and euthanized to collect the lungs, brains, and spleens aseptically for the detection of the NiV load. After the hamsters were infected with NiV-M, the viral load in the lung tissues of the control group was as high as 1.36 × 10^10^ copies/gram and 1 × 10^5^ median tissue culture infectious dose (TCID_50_)/gram, while no viral nucleic acid (Figure 6A) or infectious viral particles (VPs) (Figure 6B) were detected in the vaccinated hamsters. Similarly, no viral nucleic acid or infectious VPs were detected in the brain tissues and spleens of the vaccine regimen–immunized hamsters. In contrast, all hamsters in the empty vector control groups showed a high viral load and infectious VPs in the spleen (8.26 × 10^9^ copies/gram, 1 × 10^4.18^ TCID_50_/gram) and brain (5.22 × 10^8^ copies/gram, 1 × 10^4.62^ TCID_50_/gram), as shown in Figure 6A and Figure 6B. Likewise, after NiV-B infection, no viral nucleic acid (Figure 6C) or infectious VPs (Figure 6D) were detected in the lung, brain, or spleen tissues of the vaccine regimen–immunized hamsters, while most of the animals in the empty vector control groups, except for 2 of AdC68 in the brain, 1 of pVAX1 in the brain, 2 of pVAX1/AdC68 in the brain, 1 of AdC68 in the spleen, and 1 of pVAX1 in the spleen, showed a high viral load (Figure 6C) and infectious VPs (Figure 6D) in the lung, brain, and spleen tissues. The vaccine regimens completely protected the hamsters from infection with both NiV-M and NiV-B strains.

### No or slight pathological damage was observed in the lungs of vaccine regimen–immunized hamsters against NiV-M and NiV-B challenge.

**Figure 7:**
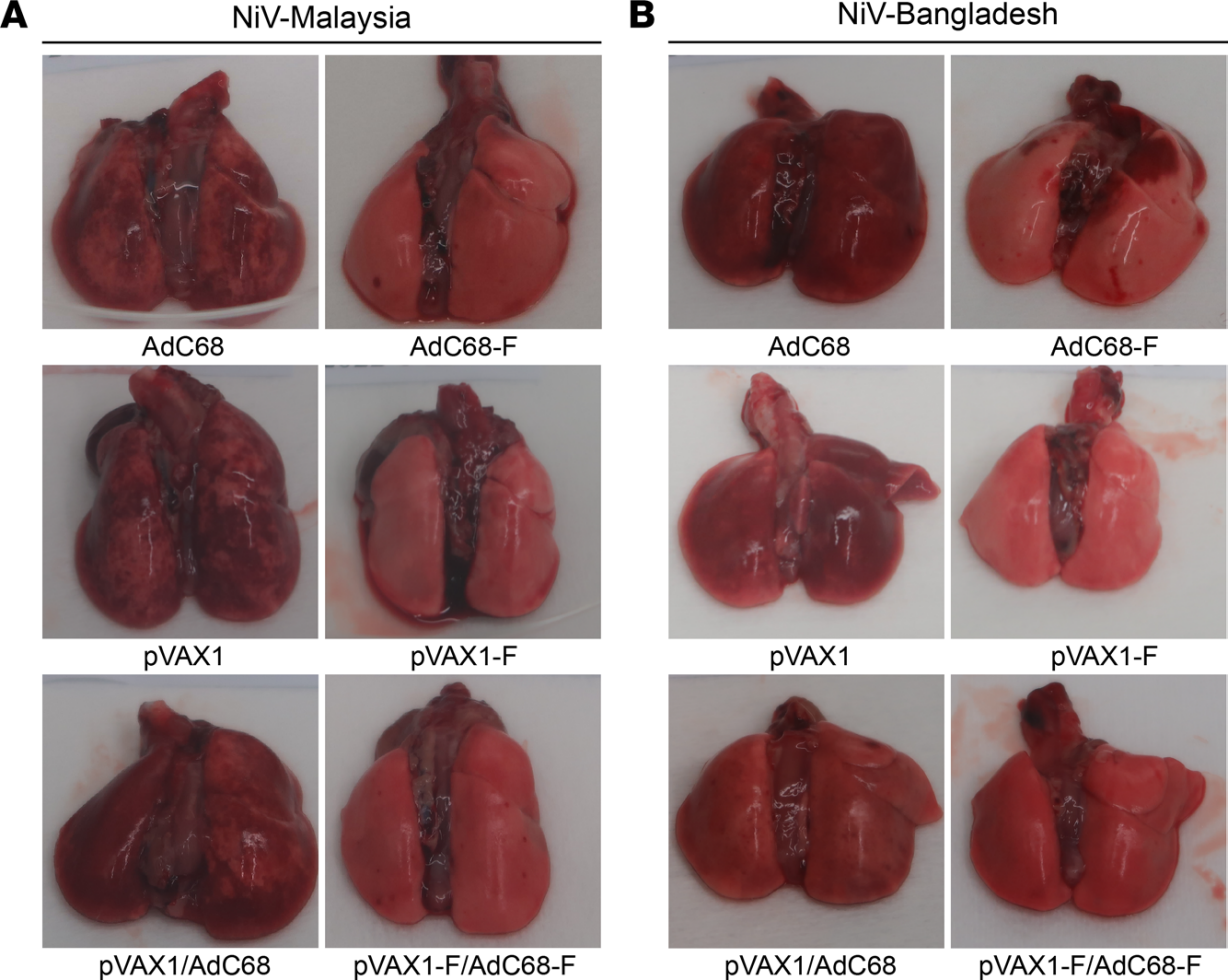
Gross pathological damage of the lungs after hamsters were challenged with NiV. NiV-M (**A**) or NiV-B (**B**) infection in empty vector–immunized hamsters (*n* = 6) resulted in marked congestion, edema, and diffuse hemorrhagic areas in the lungs, which extended to multiple lobes or even the entire lungs. However, vaccinated hamsters (*n* = 6) exhibited no or slightly abnormal pathological changes in the lungs.

Five days after NiV infection, 6 hamsters from each group were randomly selected and euthanized to examine the pathological changes in the lung and spleen tissues. The NiV-M (Figure 7A) or NiV-B (Figure 7B) infection in empty vector–immunized hamsters resulted in marked congestion, edema, and diffuse hemorrhagic areas in the lungs, which extended to multiple lobes or even the entire lungs. In contrast, vaccinated hamsters exhibited no abnormal pathological changes in the lungs or only scattered pinpoint hemorrhagic spots, with the most severe pathological changes involving only a few lung lobes. Compared with the empty vector control hamsters, the lung pathological damage was significantly reduced in the vaccinated hamsters.

**Figure 8:**
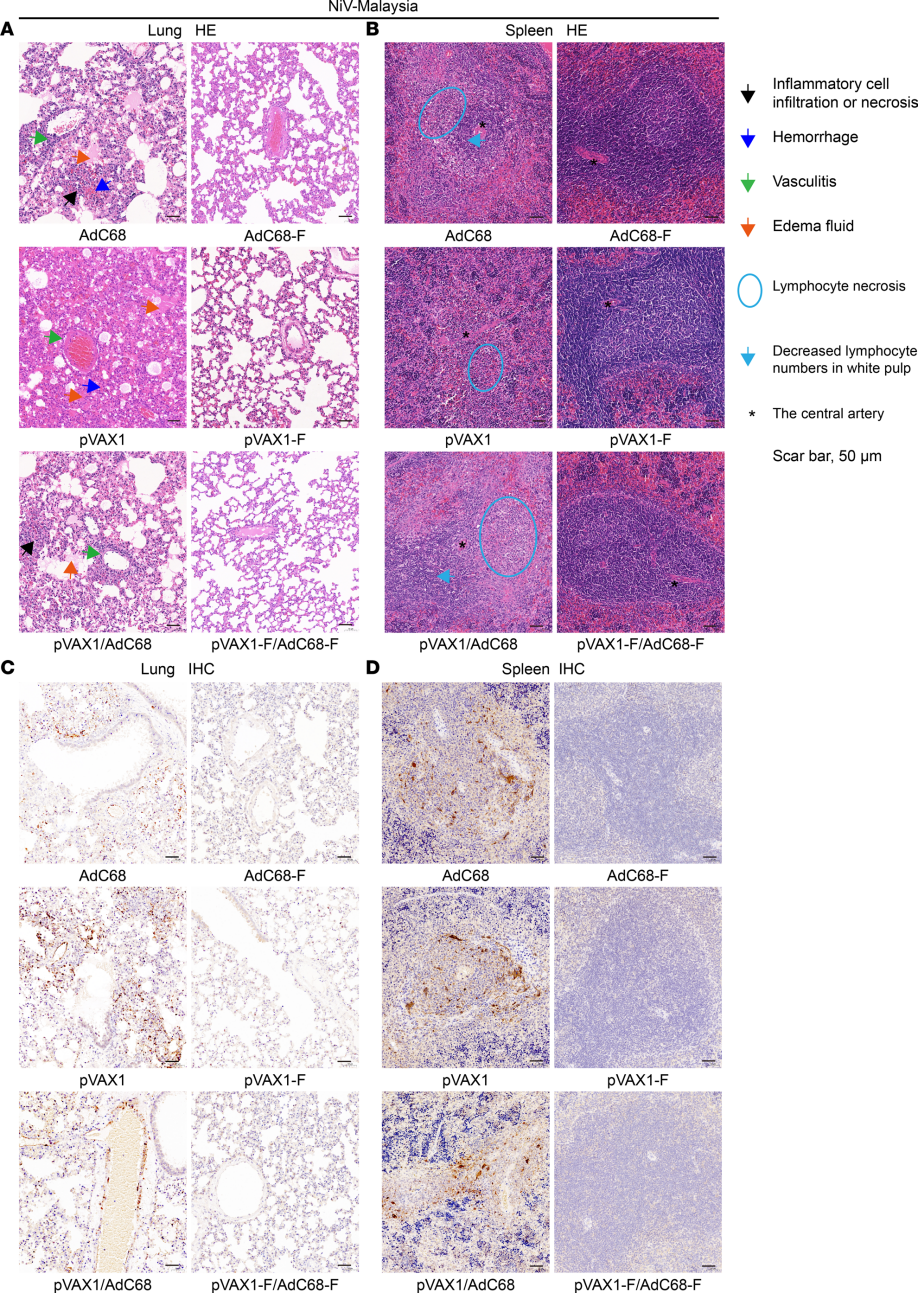
H&E and IHC staining of lungs and spleens after challenge with NiV-M. (**A**) The lung of control hamsters exhibited hemorrhage within the alveolar walls and alveoli, extensive infiltration of lymphocytes and mononuclear macrophages, and significant inflammatory reactions in blood vessels and pleura, with edema fluid and fibrinous exudates in the alveoli. The lungs of vaccine regimen hamsters showed no significant histopathological abnormalities. (**B**) Lymphocytes in the spleens of control hamsters showed single-cell necrosis or focal necrosis, and there was a decrease in white pulp lymphocytes. However, only a few hamsters in the vaccine group showed a reduction in white pulp lymphocytes. (**C**) A large amount of NiV antigen was present in the lungs of nonimmunized control hamsters, while little or no NiV antigen was detected in the lungs of vaccinated hamsters. (**D**) A large amount of NiV antigen was present in the spleens of nonimmunized control hamsters, while no NiV antigen was detected in the spleens of vaccinated hamsters. Symbols corresponding to the distinct pathological lesions and signs are indicated on the right. Scale bars: 50 μm.

**Figure 9:**
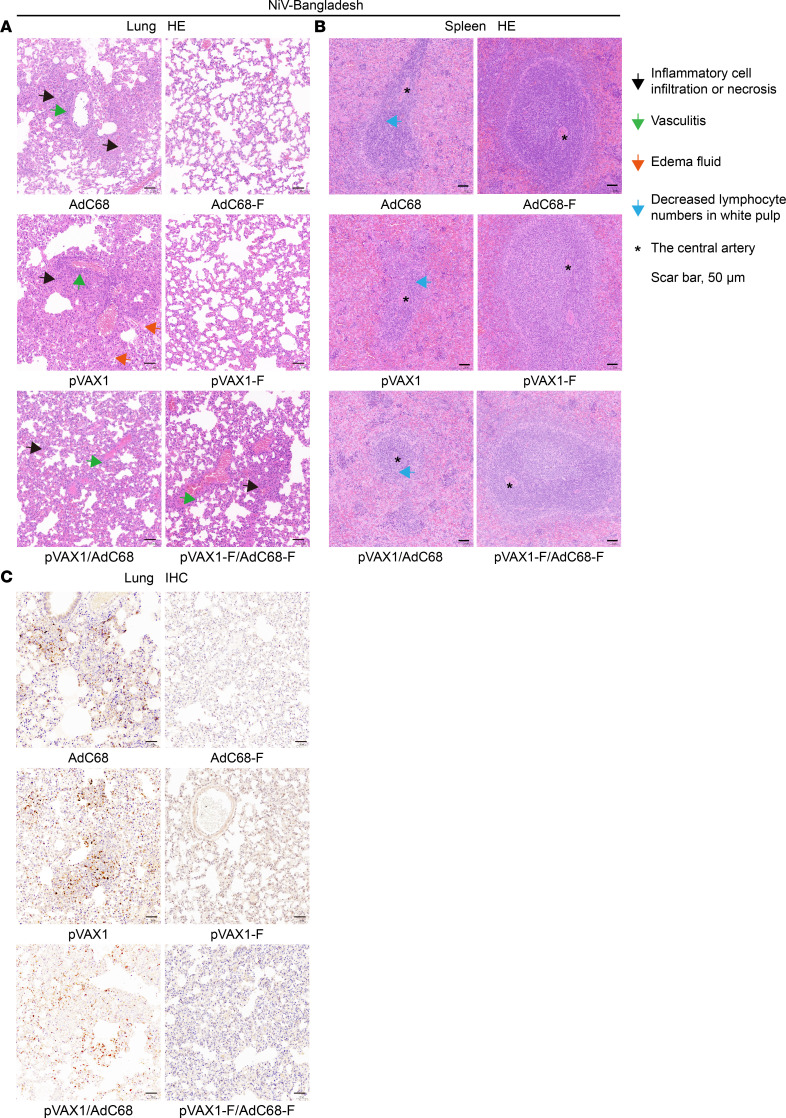
H&E and IHC staining of lungs and spleens after challenge with NiV-B. (**A**) The lung of control hamsters exhibited hemorrhage within the alveolar walls and alveoli, extensive infiltration of lymphocytes and mononuclear macrophages, and significant inflammatory reactions in blood vessels and pleura, with edema fluid and fibrinous exudates in the alveoli. The lungs of vaccine regimen hamsters showed no significant histopathological abnormalities. (**B**) Lymphocytes in the spleens of control hamsters showed single-cell necrosis or focal necrosis, and there was a decrease in white pulp lymphocytes. However, only a few hamsters in the vaccine group showed a reduction in white pulp lymphocytes. Symbols corresponding to the distinct pathological lesions and signs are indicated on the right. (**C**) A large amount of NiV antigen was present in the lungs of nonimmunized control hamsters, while little or no NiV antigen was detected in the lungs of vaccinated hamsters. Scale bars: 50 μm.

To further clarify the effectiveness of the vaccine regimen–induced immune protection, the lungs and spleens of hamsters euthanized 5 days after NiV infection were subjected to H&E staining and IHC analysis. Regardless of NiV-M or NiV-B infection, the lungs of pVAX1-, AdC68-, and pVAX1/AdC68–immunized control hamsters exhibited hemorrhage within the alveolar walls and alveoli, extensive infiltration of lymphocytes and mononuclear macrophages, and significant inflammatory reactions in blood vessels and pleura, with edema fluid and fibrinous exudates in the alveoli. In contrast, the lungs of AdC68-F–vaccinated hamsters showed no significant histopathological abnormalities. No significant histopathological abnormalities were observed in the pVAX1-F–vaccinated hamsters. Most of the pVAX1-F/AdC68-F–vaccinated hamsters showed no abnormalities, with only 1 hamster exhibiting mild vasculitis (Figure 8A and Figure 9A).

In the NiV-M or NiV-B control group, lymphocytes in the spleen showed single-cell necrosis or focal necrosis, and there was a decrease in white pulp lymphocytes. However, in the vaccinated group, only a reduction in white pulp lymphocytes was observed, which was significantly reduced compared with the nonimmunized control group (Figure 8B and Figure 9B).

Consistent with the H&E staining results, IHC analysis showed that after NiV-M or NiV-B infection, a large amount of NiV antigen was present in the lungs (Figure 8C and Figure 9C) and spleens (Figure 8D) of nonimmunized control hamsters, while no NiV antigen was detected in the lungs and spleens (NiV-B infection results not shown) of vaccinated hamsters.

In summary, both gross specimen and pathological staining results demonstrate that the vaccine effectively protects hamsters from the pathological damage caused by NiV-M or NiV-B infection.

## Discussion

NiV is a zoonotic virus that naturally infects fruit bats of the genus *Pteropus* and transmits to humans directly or through intermediate hosts, leading to person-to-person transmission (41). Due to the high likelihood of contact between intermediate hosts such as horses, pigs, dogs, and humans, the risk of NiV infection in humans is much higher than that of other viruses with wild animals as intermediate hosts. Currently, there are no effective clinical prevention and control measures for NiV disease, and vaccines are considered one of the most effective preventive measures against NiV infection. To date, multiple NiV vaccines are still in the laboratory developmental stage.

According to the search results on the ClinicalTrials.gov website, there are 3 kinds of NiV vaccines currently in the clinical stage (42). The fastest-progressing vaccine is the soluble G protein vaccine of the Hendra virus against NiV infection, sponsored by Auro Vaccines LLC. It has completed phase I clinical development as of May 6, 2022. The other 2 kinds of vaccines are an mRNA vaccine and a viral vector vaccine. mRNA-1215 encodes the secreted prefusion-stabilized F component covalently linked to the G monomer (pre-F/G) of NiV-M. PHV02 consists of vesicular stomatitis virus (VSV) with the gene for the Zaire ebolavirus glycoprotein replacing the G gene for VSV. In addition, the G protein of NiV is also inserted and expressed. However, all of these vaccines were developed against a single strain of NiV. As NiV is an RNA virus that can be transmitted through the respiratory tract, it is prone to mutation when widely spread among populations, as seen in the experience with COVID-19. Therefore, it is urgent to proactively develop an NiV vaccine that can prevent multiple NiV strain infections.

In this study, we used bioinformatics methods based on the principle of sequence consistency to design a broad-spectrum vaccine targeting the NiV fusion protein F. The recombinant chimpanzee adenoviral vector vaccine and DNA vaccine were developed after using the chimpanzee adenovirus or plasmid as a carrier. Viral vector vaccines can induce effective humoral and cellular immune responses and have been widely used in many vaccine studies (43). However, safety and preexisting immune responses have always been concerns. The chimpanzee adenoviral vector used in this study is a nonreplicating viral vector vaccine that can replicate and proliferate only in HEK293 cells with stable E1 gene expression and cannot replicate and proliferate in normal human bodies, ensuring the safety of the vaccine. Additionally, the ChAdOx1-nCoV-19 vaccine from Oxford-AstraZeneca approved by the European Medicines Agency (EMA) and WHO to prevent COVID-19 also uses a similar nonreplicating chimpanzee adenoviral vector as the carrier (44–46). One of the major advantages of this type of vaccine vector is that it avoids the possibility of preexisting antibodies in the human body. Although adenoviruses have a broad host range (47), they have strict host specificity, and adenoviruses that infect chimpanzees do not generally infect humans, thereby ensuring that the human population has no preexisting antibodies against the chimpanzee adenovirus and reducing the possibility of a decrease in vaccine efficacy. To detect the impact of preexisting antibodies on vaccine efficacy, we tested the immune response and protection effect induced by 2 doses of AdC68-F. However, such an experimental design cannot fully simulate the presence of preexisting antibodies in the human body. In future experiments, animals can be immunized with the AdC68 empty vector first and then vaccinated with the AdC68-F vaccine after detecting antibodies. The immune response and protection obtained through this approach can be used to reiterate the possible impact of preexisting immunity on vaccine efficacy.

In this study, there was no apparent benefit to PVAX1-F priming followed by AdC68-F boosting as compared with PVAX1-F immunization alone. The lack of superiority of the heterologous prime-boost regimen seems unexpected compared with other studies (48, 49), in which the initial DNA vaccine immunization was delivered i.m. and boosted by recombinant adenoviral vaccines delivered i.m. We thought the DNA vaccine delivered by i.m. injection and an additional electroporation (EP) in our study might be the key factors in the results. As we know, one of the main reasons for DNA vaccines’ poor performance is thought to be insufficient antigenic load caused by inefficient expression of the target antigenic proteins in immunologically relevant cells and tissues (50). To overcome the inefficient expression of the target antigen, EP is one of the most effective solutions. Histological analysis of the site of injection revealed that the transfection efficiency was greatly improved and the variability between animals was reduced when the DNA vaccines were delivered by EP. Additionally, other studies showed that the humoral and cellular immune responses induced by the DNA vaccine delivered by EP are not lower than, or even higher than, those of the protein vaccine delivered with adjuvant (51). As a result, since DNA vaccines are already capable of inducing a strong immune response, the superiority of adenoviral vaccine booster immunization would be greatly diminished.

The DNA vaccine developed in this study has shown high immunogenicity and complete immune protection in hamsters. However, 2 major issues have persisted in the application of DNA vaccines: the administration mode of the vaccine and the potential difference in immune protection efficacy between large animals and rodents. Further experimentation is needed to verify its efficacy in large animals such as African green monkeys. The administration method of the DNA vaccine in this study was muscle EP, which yielded promising results. However, this administration method is not suitable for potential human use. Fortunately, there have been attempts to use various other methods, such as cupping and microneedle patches, for DNA vaccine administration, which could be used for human application in the future of the developed DNA vaccine in this study (52, 53).

In previous studies (54, 55), dosages of NiV-B equal to 5.3 × 10^5^ TCID_50_ and NiV-M equal to 6.8 × 10^4^ TCID_50_ were challenged in the NiV infection model. The results showed that no virus was detected in the lung of 1 out of 4 in the control group and the brain tissue of 8 out of 8 in the control group when hamsters were challenged with NiV-B. In the same case, no virus was detected in brain tissue of 1 out of 2 in the control group when hamsters were challenged with NiV-M. Similar results showed that NiV was not detectable in the brain and lung tissues of a portion of negative control hamsters in other research groups (56). In that study, 2.23 × 10^4^ and 8.55 × 10^3^ TCID_50_ (1000 LD_50_) of NiV-B and NiV-M strains were used for the challenge study, respectively. The dosage is lower than in the current study, especially in the NiV-B strain. In our study, we did not detect the virus in some tissues in 1 or 2 hamsters in the control group challenged with NiV-B (2 AdC68 in the brain, 1 pVAX1 in the brain, 2 pVAX1/AdC68 in the brain, 1 AdC68 in the spleen, and 1 pVAX1 in the spleen). So, we speculated that when the challenge dosages were increased, the results of the virus that could not be detected in the tissues of control animals might improve. Whatever the results, they had no bearing on the fact that our vaccine fully protected hamsters against NiV.

Although the 3-vaccine regimens provided complete protection against infection by the 2 representative strains of NiV in our study, further validation is needed to verify the neutralization spectrum and immune protection against more NiV strains by packaging pseudoviruses. Furthermore, live virus challenge studies should be conducted using more NiV strains and even Hendra virus strains, where conditions permit.

In summary, this study developed a potential broad-spectrum NiV vaccine, AdC68-F and pVAX1-F, which demonstrated high immunogenicity and complete protection against NiV-M or NiV-B infection in rodent animals. After verifying its broad-spectrum immune protection in large animals, this vaccine has the potential to be developed for human use.

## Methods

### Cells and viruses.

HEK293T (ATCC, ACS-4500), HEK293 (ATCC, CRL-1573), and Vero E6 (ATCC, CRL-1586) cells were cultured in Dulbecco’s modified Eagle’s medium (DMEM) (Gibco) supplemented with 10% heat-inactivated fetal bovine serum (FBS), 1 mM L-glutamine, 100 international units (IU)/mL penicillin, and 100 μg/mL streptomycin, and cultured at 37°C in 5% CO_2_. The NiV-M (GenBank ID: AF212302.2; https://www.ncbi.nlm.nih.gov/genbank/) and NiV-B (GenBank ID: AY988601.1) strains employed in the challenge studies were obtained from the National Virus Resource, Wuhan Institute of Virology, Chinese Academy of Sciences. All virus propagation was performed in Vero E6 cells cultured in DMEM supplemented with 2% FBS.

### The NiV F protein consensus sequence designed by bioinformatics analysis.

Seventy-seven sequences of F protein were downloaded from GenBank, followed by sequence alignment and duplicate removal. Fourteen of them (GenBank numbers AY988601.1, MK673568.1, JN808864.1, MK673580.1, MK673584.1, MK673588.1, FJ513078.1, MH523641.1, MN549407.1, MN549408.1, MK801755.1, AJ627196.1, AF212302.2, and MW535746.1) were left for further consensus analysis to create the consensus sequence with the function “cons” in EMBOSS software (https://www.ebi.ac.uk/Tools/emboss/) according to the sequence consistency principle. To understand the conservation of the consensus sequence, an unrooted phylogenetic tree was constructed by the maximum likelihood method in Mega11 (https://www.megasoftware.net/). Next, the structure of the designed F protein was analyzed online by SWISS-MODEL analysis (https://swissmodel.expasy.org/). The acquired 3D model was conducted by the website automatic analysis and choice of the crystallographic prefusion F protein (PDB: 5EVM) as the template. Analysis of homology between them was conducted by PyMOL software (https://pymol.org/2/).

### Generation of vaccine AdC68-F.

The full-length F sequences used in this study were synthesized by GenScript and cloned into the transgene expression plasmid pShuttle2 comprising a human cytomegalovirus (CMV) immediate early promoter and the polyadenylation signal from bovine growth hormone (BGH). Subsequently, after digestion with *I-Ceu*I and *PI-Sce*I restriction enzymes, the whole cassette was inserted into the E1-deleted region of the chimpanzee adenoviral vector pAdC68, provided by Dongming Zhou (Tianjin Medical University, Tianjin, China) to generate the recombinant plasmid pAdC68-F. The resultant pAdC68-F, as well as empty control pAdC68, were linearized by *Pac*I and transfected into HEK293 cells to rescue recombinant viruses AdC68-F and AdC68. After the viruses were successfully rescued in HEK293 cells, CsCl gradient ultracentrifugation was conducted for purified AdC68-F and AdC68. After being titrated by spectrophotometry, the recombinant viruses were stored at −80°C before use.

### NiV DNA vaccine construction.

The optimized full-length F gene was subcloned into the clinically used vector pVAX1 between the restriction sites *Kpn*I and *BamH*I with a Kozak sequence incorporated at the 5′ end of the genes to create the optimized pVAX1-F DNA vaccine. All of the DNA vaccine plasmids, both pVAX1-F and empty vector pVAX1, were transformed into competent Top 10 bacteria for amplification. Plasmids were extracted and purified using the Endo-Free Plasmid Plus Maxi Kit (Qiagen, 12963) according to the manufacturer’s protocol.

### Western blot analysis.

HEK293 cells propagated in 6-well tissue culture plates (NEST) were infected with AdC68-F or AdC68. HEK293T cells in 6-well plates were transfected with 2 μg of pVAX1-F or empty pVAX1 using Lipofectamine 2000 (Invitrogen, 11668019) according to the manufacturer’s directions. Forty-eight hours after infection or transfection, the cells were harvested and lysed with 150 μL of RIPA lysis buffer containing a protease inhibitor cocktail (Roche, 04693159001). Cell extracts were mixed 3:1 with 4× Laemmli buffer (Bio-Rad, 1610747) plus β-mercaptoethanol and heated at 70°C for 10 minutes. Next, cell extracts were separated by electrophoresis in a 10% polyacrylamide gel and transferred to a polyvinylidene fluoride (PVDF) membrane (Millipore, IPVH00010). The membranes were blocked with 5% skim milk in Tris-buffered saline with Tween 20 (TBST). The F antigens were detected using a polyclonal rabbit anti–NiV glycoprotein F0 antibody (AntibodySystem, PVV08101; diluted 1:5000). The control antigen was detected using a monoclonal mouse anti-GAPDH antibody (Abcam, ab8245; diluted 1:5000). Anti-mouse or anti-rabbit horseradish peroxidase–conjugated (HRP-conjugated) antibody (Proteintech, SA00001-1; diluted 1:2000) was used as a secondary antibody. Bands were visualized using an enhanced chemiluminescence detection kit following the manufacturer’s instructions (Thermo Fisher Scientific, 34577).

### Animal experiments.

Six- to 8-week-old female BALB/c mice and 5- to 6-week-old female Syrian hamsters were purchased from Vital River Laboratories. BALB/c mice were randomly allocated into 8 groups (Table 1). For DNA vaccine (pVAX1-F and pVAX1) immunization, anesthetized BALB/c mice were injected i.m. with 50 μL of solution containing the indicated DNA. Following instillation, the injection site was subjected to EP at a constant voltage of 100 V, with 3 pulses at 50 msec per pulse and 1-second intervals between pulses. Electrical pulses were delivered by an electric pulse generator (BTX). The AdC68-F vaccine was administered by i.m. injection. The mice in group 2 (*n* = 12) were vaccinated with AdC68-F at 5 × 10^10^ VPs, and the mice in group 4 (*n* = 12) were vaccinated twice with 5 × 10^10^ VPs of AdC68-F at a 3-week interval. The mice in group 6 (*n* = 18) were vaccinated with 50 μg of pVAX1-F on day 0 and AdC68-F on day 21. The mice in group 8 (*n* = 24) were vaccinated with 50 μg of pVAX1-F on days 0, 21, and 42. Groups 1 (*n* = 6), 3 (*n* = 6), 5 (*n* = 9), and 7 (*n* = 12) were given the corresponding empty vector AdC68 or pVAX1 as a control. On day 10 after each vaccination, mice (*n* = 3 per control group, *n* = 6 per vaccine group) were sacrificed, and splenocytes were harvested for the detection of cellular immune responses. Sera were collected at predetermined time intervals for the detection of humoral immune responses (Figure 3A).

Hamsters were randomly divided into 6 groups (Table 2). Two groups (groups 1 and 2) of animals were homologously vaccinated with 5 × 10^10^ VPs of AdC68-F (*n* = 12) or AdC68 (*n* = 9) via an i.m. route in a 3-week interval. Two groups (groups 3 and 4) of animals were prime vaccinated with 100 μg of pVAX1-F (i.m./EP, *n* = 12) or pVAX1 (*n* = 9) and boosted with 5 × 10^10^ VPs of AdC68-F or AdC68 via the i.m. route in a 3-week interval. Two groups (groups 5 and 6) of animals were immunized 3 times with 100 μg of pVAX1-F (i.m./EP, *n* = 12) or pVAX1 (*n* = 9) on days 0, 21, and 42. For DNA vaccine immunization in hamsters, the injection site was electroporated immediately after instillation (100 μL per animal) at a constant 75 V, with 10 pulses at 50 msec per pulse and 100 msec intervals between pulses. Serum samples were collected at the indicated time points and analyzed by immunological assays (Figure 4A). Two weeks after the last vaccination, all animals were transferred to the ABSL-4. After a 1-week adaptation, hamsters challenged with either 1000 LD_50_ of NiV-M or NiV-B strains in 500 μL of DMEM via the i.p. injection route. Six animals in each group were euthanized 5 days after infection, and lung, brain, and spleen tissues were harvested and subjected to virological and histological analysis. The remaining animals (*n* = 3 per control group, *n* = 6 per vaccine group) were monitored daily for signs of disease, and their weights were recorded up to 21 days after challenge.

### ELISA.

Serum was harvested from individual mice at different weeks after immunization, as well as from individual hamsters at 3 weeks after each vaccination. NiV F protein (AntibodySystem, EVV08101) was diluted in coating buffer (Solarbio, C1055) at a concentration of 2 μg/mL and then plated in high-bind ELISA plates (Corning) at 100 μL in each well. After overnight incubation at 4°C, plates were washed twice with PBS, blocked with PBS plus 5% skim milk, and incubated for 1 hour at 37°C. Plates were then washed twice with PBS and incubated for 2 hours at 37°C with 2-fold serial dilutions of sera in PBS containing 5% skim milk (100 μL/well). Plates were washed 4 times with PBST (0.05% Tween 20 in 1× PBS), followed by the addition of HRP-conjugated goat anti-mouse (Abcam, ab6789; diluted 1:20000) and HRP-conjugated goat anti-hamster IgG (Abcam, ab6892; diluted 1:15000) diluted in 5% milk in PBS for 1.5 hours at 37°C. Plates were washed 4 times with PBST before being developed with 100 μL/well of TMB chromogen solution (Beyotime, P0209) for 15 minutes. The reaction was stopped by the addition of 50 μL of stop solution for TMB (Beyotime, P0215) per well. The optical density (OD) was determined at 450 nm. Endpoint titers were calculated as the dilution that emitted an OD exceeding background.

### ELISpot assay.

The ELISpot assay was performed with a mouse IFN-γ ELISpot kit (Mabtech, 3321-4APT-10) to detect the T cell response induced by the vaccine regimens. The spleens were harvested 10 days after each vaccination. Single-cell suspensions were collected after passing through a 70-μm cell strainer (Falcon, 352350). Splenocytes were then stimulated with 2 NiV F protein peptide pools (18-mers with a 10–amino acid overlap). The final concentration of each peptide was 2 μg/mL. Positive control wells were stimulated with 500 ng/mL phorbol 12-myristate 13-acetate and 10 μg/mL ionomycin (Dakewe, 2030421), whereas the negative control wells received no stimuli. The cells were then incubated with 5% CO_2_ at 37°C for 24 hours. Next, a biotinylated detection antibody and streptavidin-HRP were added. Blots were developed by the addition of the substrate 3-amino-9-ethylcarbazole (AEC), which produced a colored spot after 5 minutes of room temperature incubation in the dark. Finally, IFN-γ^+^ SFCs were visualized on a Cellular Technology Ltd. imager, and counting was performed with the included ImmunoSpot software.

### Pseudovirus neutralization assay.

The NiV pseudovirus was generated by cotransfection of HEK293T cells with the Env-defective, luciferase-expressing HIV-1 backbone (pNL4-3.Luc-R-E-), and pcDNA3.1 encoding the F25 (1–523 aa, the sequence used in this study) and G33 (34–602 aa, the consensus sequence of G obtained by the bioinformatics analysis method used in this study) proteins of NiV. Forty-eight hours later, the supernatants containing the pseudovirus were collected, filtered, and stored below −80°C. Serum samples were heat inactivated at 56°C for 30 minutes and serially diluted 3-fold in 96-well cell culture plates before NiV pseudovirus was added and incubated at 37°C for 1 hour. HEK293T cells (3 × 10^4^) were seeded on a serum pseudovirus mixture. After 48 hours, the activity of luciferase was measured using a Luciferase Assay (Promega, E2620). The NA efficiency was calculated as follows: (relative luciferase units of mock sera – relative luciferase units of immune serum for a given dilution)/relative luciferase units of mock sera. The NA titers based on NiV pseudovirus is defined as the serum dilution when the NA efficiency is greater than 50%.

### Microneutralization assay.

For the live virus neutralization assays, the serum samples (heat inactivated for 30 minutes at 56°C) harvested from hamsters were serially diluted 3-fold from an initial 1:20 dilution of each sample in DMEM containing 2% FBS. The samples were then added to either 100 TCID_50_ of NiV-M or 50 TCID_50_ of NiV-B and subsequently incubated at 37°C for 1 hour. Following incubation, the virus-serum mixtures were incubated for 1 hour with Vero E6 cells at 37°C with 5% CO_2_. Quadruplicates were prepared for each serum dilution. The CPE was scored on wells until 5 dpi. The virus neutralization titer is expressed as the reciprocal of the highest dilution of the serum that prevented infection in 50% of quadruplicate inoculations.

### Quantitative real-time reverse transcriptase PCR.

A clarified supernatant of homogenized lung, brain, and spleen tissues from NiV-infected hamsters was harvested for viral load detection. RNA was extracted using the RNeasy kit (Qiagen, 52906) according to the manufacturer’s instructions, and the total RNA was eluted in 50 μL of elution buffer. RNA (2 μL) was used as a template for quantitative real-time reverse transcriptase PCR (qRT-PCR). qRT-PCR was performed on a CFX96 Real-Time System (Bio-Rad) using the HiScript II One Step qRT-PCR Probe Kit (Vazyme, Q222-01) with primers and probes specific for the NiV nucleocapsid (N) gene: forward primer (5′-AACATCAGCAGGAAGGCAAGA-3′), reverse primer (5′-GCCACTCTGTTCTATAGGTTCTTC-3′), and probe (5′-FAM-TTGCTGCAGGAGGTGTGCTC-BHQ1-3′). The standard curve was constructed with 9 points in a 20 μL reaction system (1 × 10^9^ to 1 × 10^1^ copies). Samples with fewer than 10 copies were defined as negative.

### Virus titration assay.

Viral titrations were performed in Vero E6 cells, which were inoculated with 10-fold serial dilutions of tissue homogenate (100 μL/well). After a 1-hour incubation at 37°C with 5% CO_2_, tissue homogenate dilutions were removed, and cells were washed twice with PBS and incubated in 100 μL DMEM containing 2% FBS. The virus-induced CPEs in each well were recorded at 5 days after infection, and the results are expressed as TCID_50_ according to the method of Reed and Muench (57).

### Histology and IHC.

Lung, brain, and spleen tissues of hamsters were fixed in 10% formalin for 7 days. The formalin solution was replaced twice before being transferred out of the ABSL-4 facility. Then, the samples were embedded in paraffin, sequentially sectioned to 4 μm thickness, and stained with H&E prior to examination by light microscopy. To obtain a representative result, different sites of the lung tissue and almost the whole spleen were sampled for histopathology analysis.

Specific anti-NiV immunoreactivity was detected using anti–NiV N protein rabbit primary antibodies (prepared by our laboratory) at 1:3000 dilution at 4°C overnight. The secondary antibody used was biotinylated goat anti–rabbit IgG (SeraCare, 5220-0336) at a 1:200 dilution for 50 minutes. Slides were developed with diaminobenzidine (DAB) chromogen for 15 seconds and counterstained with hematoxylin for 45 seconds. Images were acquired with a Pannoramic MIDI system (3DHISTECH Ltd.).

### Statistics.

Statistical analyses were performed with GraphPad Prism 8.0 software. Two-tailed, unpaired Student’s *t* tests were conducted to compare differences between 2 experimental groups. One-way ANOVA with Tukey’s multiple-comparison test was applied to compare more than 2 experimental groups. A *P* value of less than 0.05 was considered significant: *P < 0.05, **P < 0.01, ***P < 0.001, ****P < 0.0001. NS, not significant.

### Study approval.

All animal work was approved by the Institutional Animal Care and Use Committee (Ethics Number: WIVA21202104) of the Wuhan Institute of Virology, Chinese Academy of Sciences. All infectious work involving NiV was performed in the BSL-4 facility at the National Biosafety Laboratory, Chinese Academy of Sciences.

### Data availability.

Values for all data points shown in the figures can be found in the Supporting Data Values Excel file in the supplemental material. Other data that support the findings of this study are available from the corresponding author upon reasonable request.

## Author contributions

The order of co–first authors ML and YY was determined by their degrees of contribution. JL, CS, SC, ZY, and YY conceived and designed the research and review and edited the manuscript. JL performed the animal experiments, data analysis, acquired funding, and wrote the initial manuscript. ML generated the vaccine and performed the experiments and analyzed all data. YY performed the animal experiments and acquired funding. Xuekai Zhang, XL, TC, YS, JZ, XiaoYu Zhang, CY, WG, PY, and JR participated in mouse experiments. YL performed data analysis. YY, HL, GG, YP, XH, and MC performed BSL-4 and ABSL-4 experiments. EL and GW contributed to the investigation. All authors discussed the results and approved the manuscript.

## Supplementary Material

Supplemental data

Supporting data values

## Figures and Tables

**Table 1:**
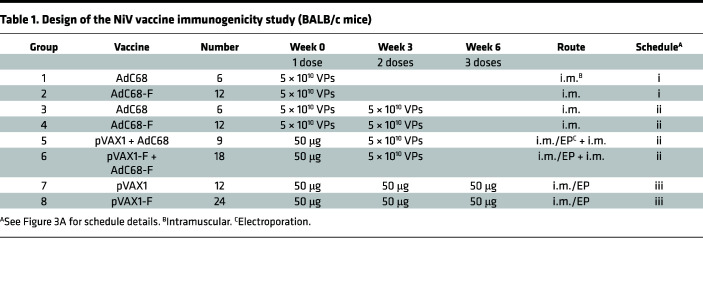
Design of the NiV vaccine immunogenicity study (BALB/c mice)

**Table 2:**
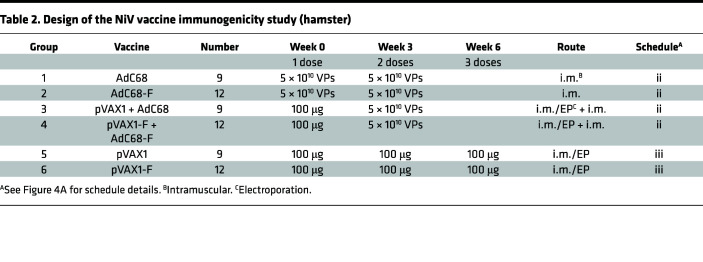
Design of the NiV vaccine immunogenicity study (hamster)

## Version 1 11/02/2023

In-Press Preview

## Version 2 12/08/2023

Electronic publication
