# Supplementary material for: Vaccines based on the fusion protein consensus sequence protect Syrian hamsters from Nipah virus infection
Source: JCI Insight. 2023 Dec 8;8(23):e175461. doi: 10.1172/jci.insight.175461 (PMC10795836; doi:10.1172/jci.insight.175461)
Supplement: Supplemental data [file jciinsight-8-175461-s155.pdf]

A

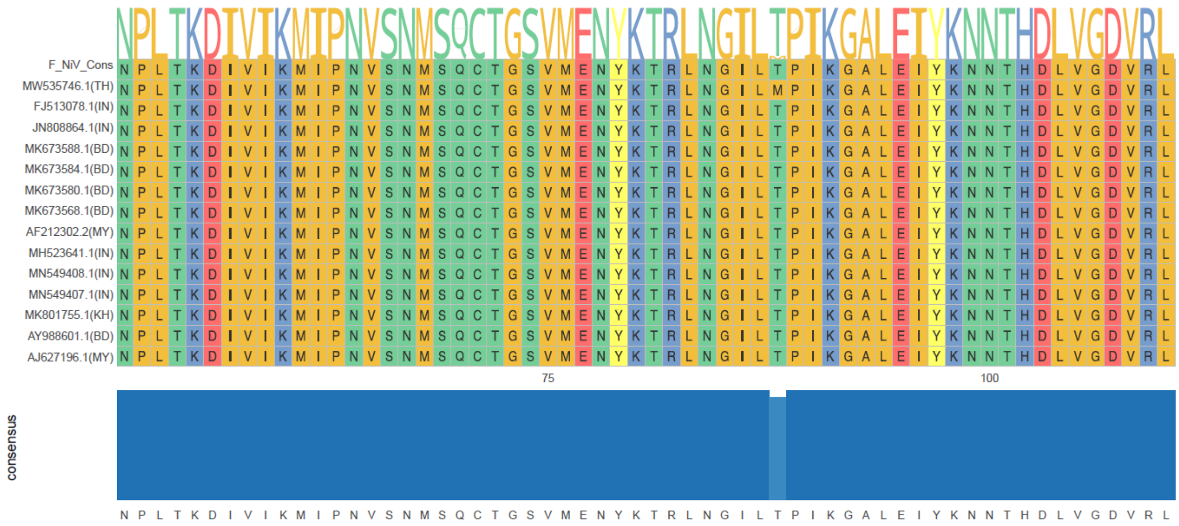
NPLTKDIVIKMIPNVSNMSQCTGSVMENYKTRLNGILTPIKGALEIYKNNTHDLVGDVRL
F_NiV_Cons NPLTKDIVIKMIPNVSNMSQCTGSVMENYKTRLNGILTPIKGALEIYKNNTHDLVGDVRL
MW535746.1(TH) NPLTKDIVIKMIPNVSNMSQCTGSVMENYKTRLNGILMPIKGALEIYKNNTHDLVGDVRL
FJ513078.1(IN) NPLTKDIVIKMIPNVSNMSQCTGSVMENYKTRLNGILTPIKGALEIYKNNTHDLVGDVRL
JN808864.1(IN) NPLTKDIVIKMIPNVSNMSQCTGSVMENYKTRLNGILTPIKGALEIYKNNTHDLVGDVRL
MK673588.1(BD) NPLTKDIVIKMIPNVSNMSQCTGSVMENYKTRLNGILTPIKGALEIYKNNTHDLVGDVRL
MK673584.1(BD) NPLTKDIVIKMIPNVSNMSQCTGSVMENYKTRLNGILTPIKGALEIYKNNTHDLVGDVRL
MK673580.1(BD) NPLTKDIVIKMIPNVSNMSQCTGSVMENYKTRLNGILTPIKGALEIYKNNTHDLVGDVRL
MK673568.1(BD) NPLTKDIVIKMIPNVSNMSQCTGSVMENYKTRLNGILTPIKGALEIYKNNTHDLVGDVRL
AF212302.2(MY) NPLTKDIVIKMIPNVSNMSQCTGSVMENYKTRLNGILTPIKGALEIYKNNTHDLVGDVRL
MH523641.1(IN) NPLTKDIVIKMIPNVSNMSQCTGSVMENYKTRLNGILTPIKGALEIYKNNTHDLVGDVRL
MN549408.1(IN) NPLTKDIVIKMIPNVSNMSQCTGSVMENYKTRLNGILTPIKGALEIYKNNTHDLVGDVRL
MN549407.1(IN) NPLTKDIVIKMIPNVSNMSQCTGSVMENYKTRLNGILTPIKGALEIYKNNTHDLVGDVRL
MK801755.1(KH) NPLTKDIVIKMIPNVSNMSQCTGSVMENYKTRLNGILTPIKGALEIYKNNTHDLVGDVRL
AY988601.1(BD) NPLTKDIVIKMIPNVSNMSQCTGSVMENYKTRLNGILTPIKGALEIYKNNTHDLVGDVRL
AJ627196.1(MY) NPLTKDIVIKMIPNVSNMSQCTGSVMENYKTRLNGILTPIKGALEIYKNNTHDLVGDVRL
75
100
consensus
N P L T K D I V I K M I P N V S N M S Q C T G S V M E N Y K T R L N G I L T P I K G A L E I Y K N N T H D L V G D V R L

B

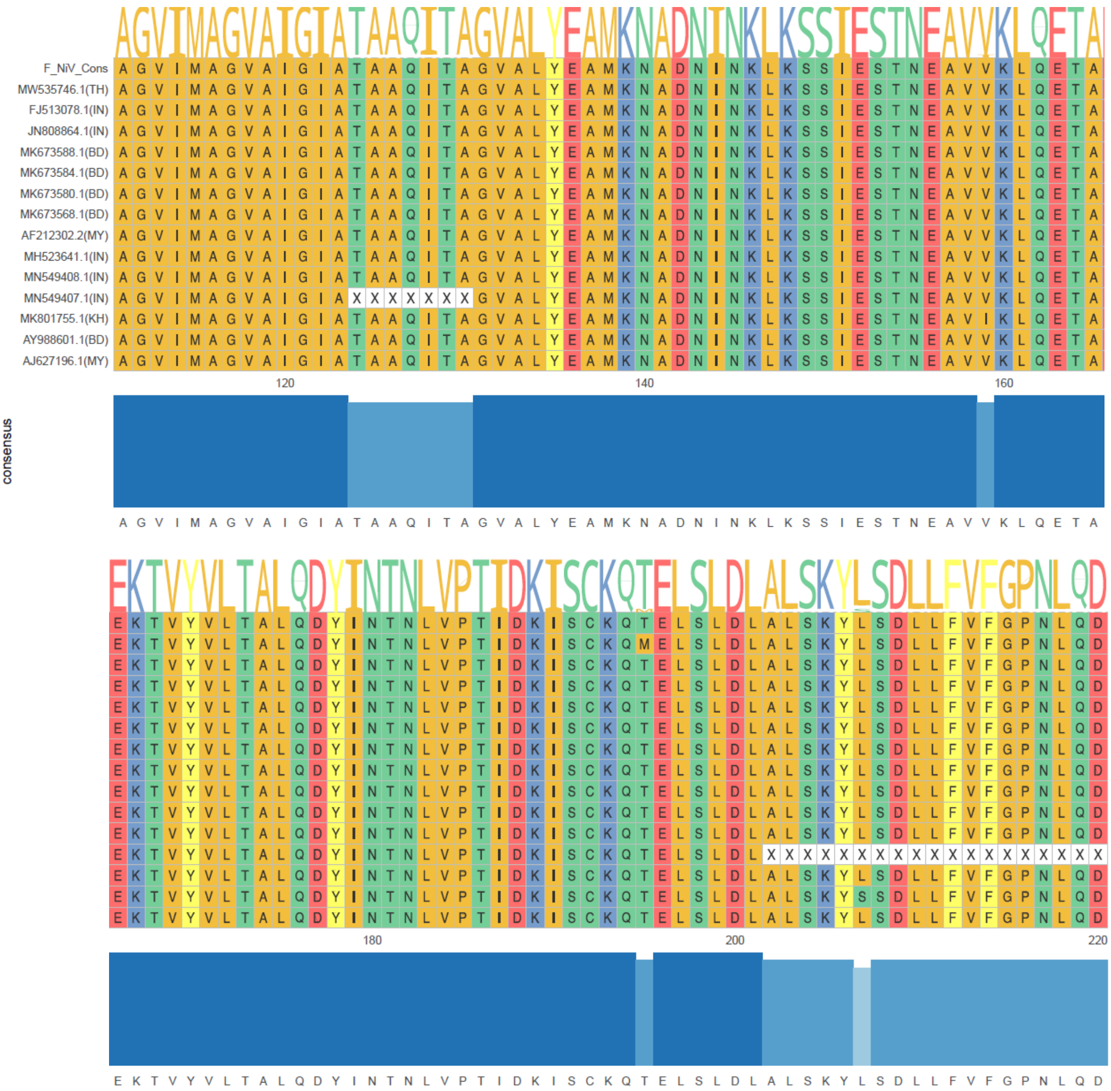

F_NiV_Cons
MW535746.1(TH)
FJ513078.1(IN)
JN808864.1(IN)
MK673588.1(BD)
MK673584.1(BD)
MK673580.1(BD)
MK673568.1(BD)
AF212302.2(MY)
MH523641.1(IN)
MN549408.1(IN)
MN549407.1(IN)
MK801755.1(KH)
AY988601.1(BD)
AJ627196.1(MY)
120
140
160
consensus
A G V I M A G V A I G I A T A A Q I T A G V A L Y E A M K N A D N I N K L K S S I E S T N E A V V K L Q E T A
180
200
220
E K T V Y V L T A L Q D Y I N T N L V P T I D K I S C K Q T E L S L D L A L S K Y L S D L L F V F G P N L Q D

C

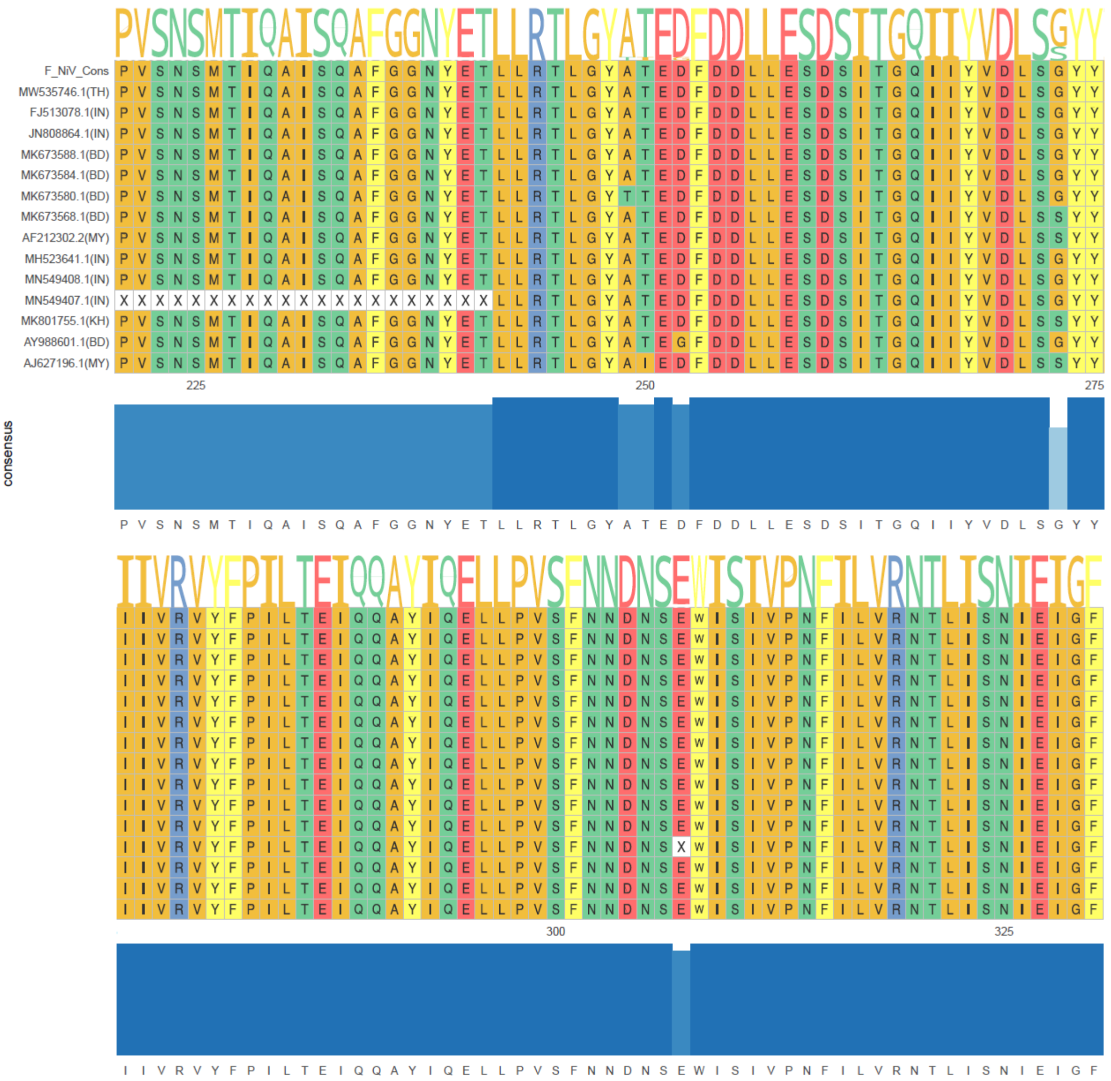

F_NiV_Cons
MW535746.1(TH)
FJ513078.1(IN)
JN808864.1(IN)
MK673588.1(BD)
MK673584.1(BD)
MK673580.1(BD)
MK673568.1(BD)
AF212302.2(MY)
MH523641.1(IN)
MN549408.1(IN)
MN549407.1(IN)
MK801755.1(KH)
AY988601.1(BD)
AJ627196.1(MY)
225
250
275
consensus
P V S N S M T I Q A I S Q A F G G N Y E T L L R T L G Y A T E D F D D L L E S D S I T G Q I I Y V D L S G Y Y
300
325
I I V R V Y F P I L T E I Q Q A Y I Q E L L P V S F N N D N S E W I S I V P N F I L V R N T L I S N I E I G F

D

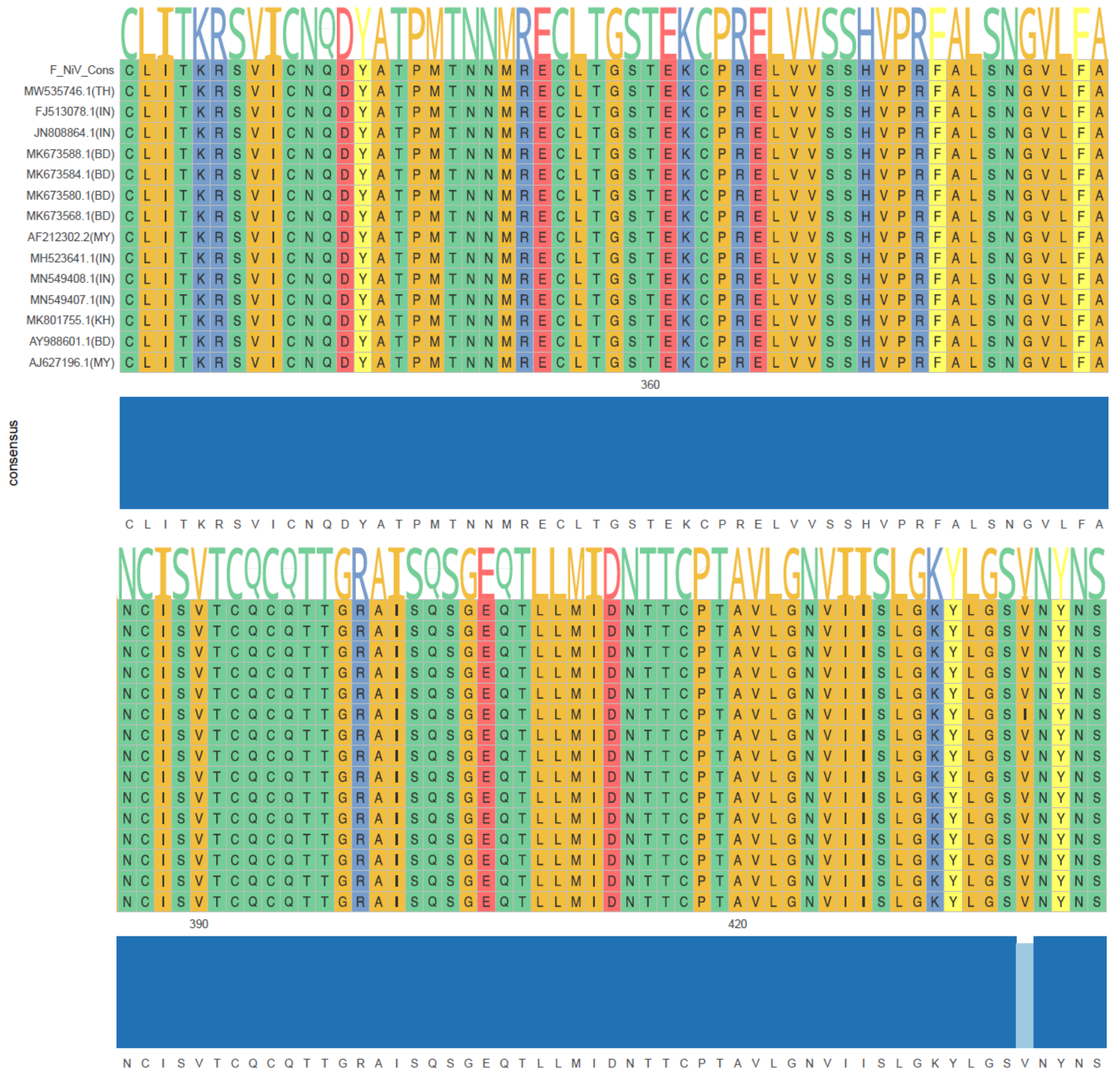

F_NiV_Cons
MW535746.1(TH)
FJ513078.1(IN)
JN808864.1(IN)
MK673588.1(BD)
MK673584.1(BD)
MK673580.1(BD)
MK673568.1(BD)
AF212302.2(MY)
MH523641.1(IN)
MN549408.1(IN)
MN549407.1(IN)
MK801755.1(KH)
AY988601.1(BD)
AJ627196.1(MY)
360
consensus
C L I T K R S V I C N Q D Y A T P M T N N M R E C L T G S T E K C P R E L V V S S H V P R F A L S N G V L F A
390
420
N C I S V T C Q C Q T T G R A I S Q S G E Q T L L M I D N T T C P T A V L G N V I I S L G K Y L G S V N Y N S

E

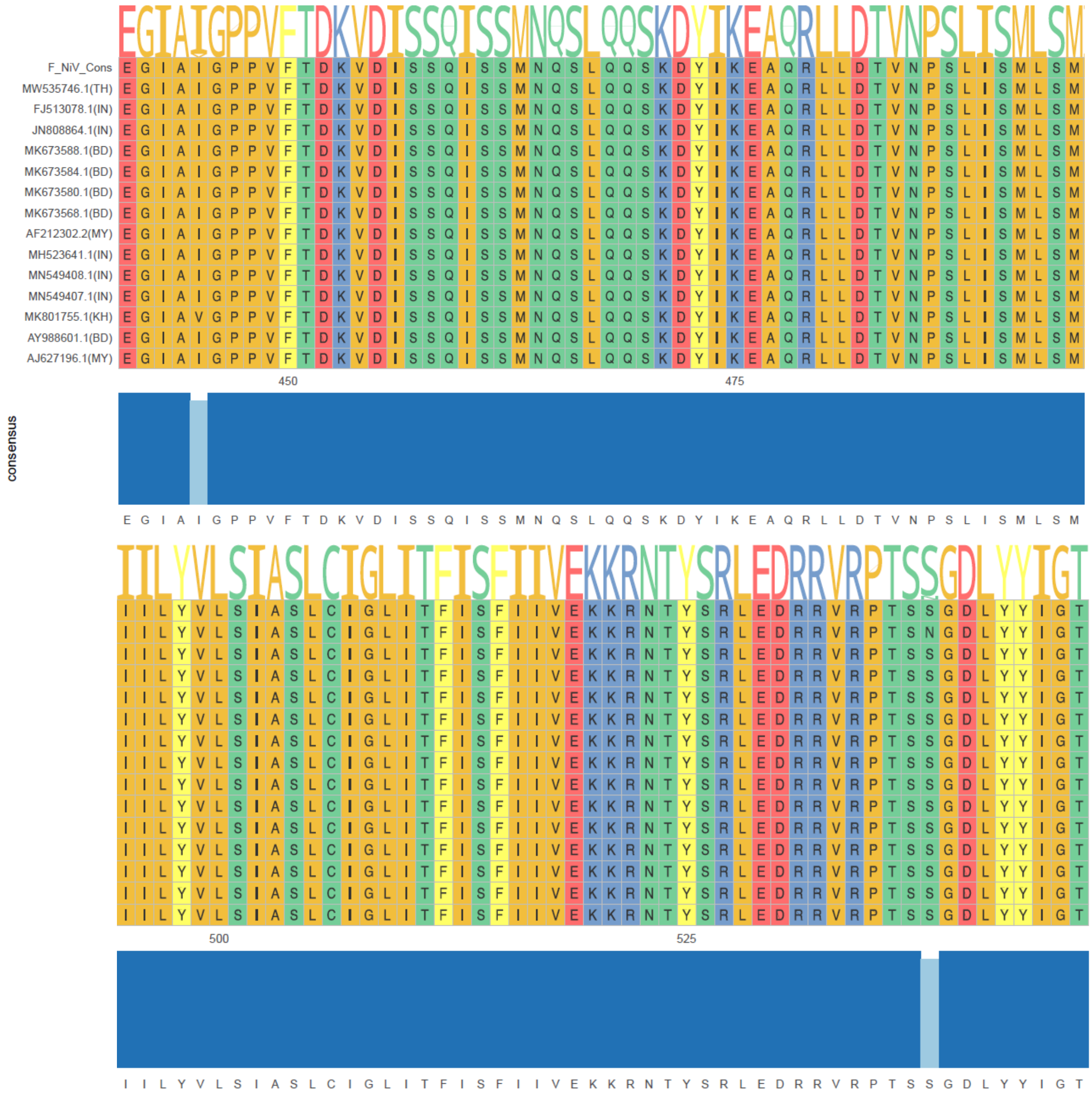

Supplemental Figure 1 The consensus sequence of the F protein in the vaccine study by bioinformatics analysis. In addition to the sequences presented in the text. (**A**) 51-110 aa, (**B**) 111-220aa, (**C**) 221-330aa, (**D**) 331-440aa, (**E**) 441-546aa. Sequences were shown by ggmsa function analysis in the R software package obtained amino acid consistency results.
